# The chemistry of isoindole natural products

**DOI:** 10.3762/bjoc.9.243

**Published:** 2013-10-10

**Authors:** Klaus Speck, Thomas Magauer

**Affiliations:** 1 Department of Chemistry, Ludwig-Maximilians-Universität München, Butenandtstraße 5–13, 81377 München, Germany

**Keywords:** isoindole, isoindoline, isoindolinone, isoindolone, natural products

## Abstract

This review highlights the chemical and biological aspects of natural products containing an oxidized or reduced isoindole skeleton. This motif is found in its intact or modified form in indolocarbazoles, macrocyclic polyketides (cytochalasan alkaloids), the aporhoeadane alkaloids, meroterpenoids from *Stachybotrys species* and anthraquinone-type alkaloids. Concerning their biological activity, molecular structure and synthesis, we have limited this review to the most inspiring examples. Within different congeners, we have selected a few members and discussed the synthetic routes in more detail. The putative biosynthetic pathways of the presented isoindole alkaloids are described as well.

## Introduction

Isoindole (2*H*-isoindole, **1**), known since more than a century, consists of a fused benzopyrrole ring system and constitutes the regioisomer of the abundant 1*H*-indole heterocycle. The fully reduced member of the isoindole family is termed isoindoline (2,3-dihydro-1*H*-isoindole, **2**). Formal oxidation to the 10π-system leads to isoindole (**1**), which is usually only stable when the labile *ortho*-quinoid structure is embedded in a π-system [1]. Incorporation of additional oxygen gives the isoindolinone (1,3-dihydro-2*H*-isoindole-1-one, **3**) and phthalimide (1,3-dihydro-2*H*-isoindole-1,3-dione, **4**) substitution pattern.

**Figure 1:**
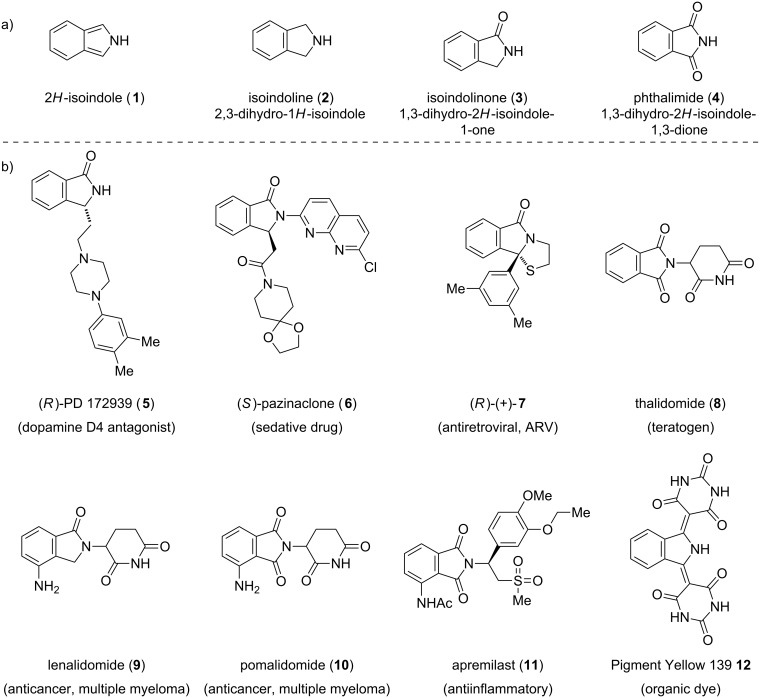
a) Structural features and b) selected examples of non-natural congeners.

The isoindole structure has attracted scientists for decades and can be found in several natural and pharmaceutical compounds [2–3]. A number of structures were explored over the years and promising drug conjugates such as **5**–**11** could be developed (Figure 1).

Compared to the synthesis of indoles, where a number of named-reactions have been reported, only conventional methods are used for the related isoindole motif. For the construction of this rare skeleton inter- and intramolecular Diels–Alder reactions are one of the most powerful methods. This was also exemplified by medicinal chemists from AstraZeneca in the manufacturing route to an mGluR2 positive allosteric modulator [4]. In 2012, a programmable enantioselective one-pot synthesis of isoindolines was reported by Waldmann [5]. Several other strategies were pursued for the synthesis of isoindoline-type structures and the synthesis, chemical and spectroscopic properties of this substance class were reviewed elsewhere [6–8].

In the late 1950s, thalidomide (**8**), a phthalimide-based drug used by pregnant women against morning sickness, became the most infamous drug in history and caused thousands of fatal casualties as well as numerous severe birth defects. Modification of the phthalimide core led to the approval of lenalidomide (**9**) [9] in 2004 and pomalidomide (**10**) in 2013 by the Food and Drug Administration (FDA) as drugs against multiple myeloma. The phosphodiesterase 4 (PDE4) inhibitor apremilast (**11**), which lacks the glutarimide is currently in phase III clinical trials.

**Scheme 1:**
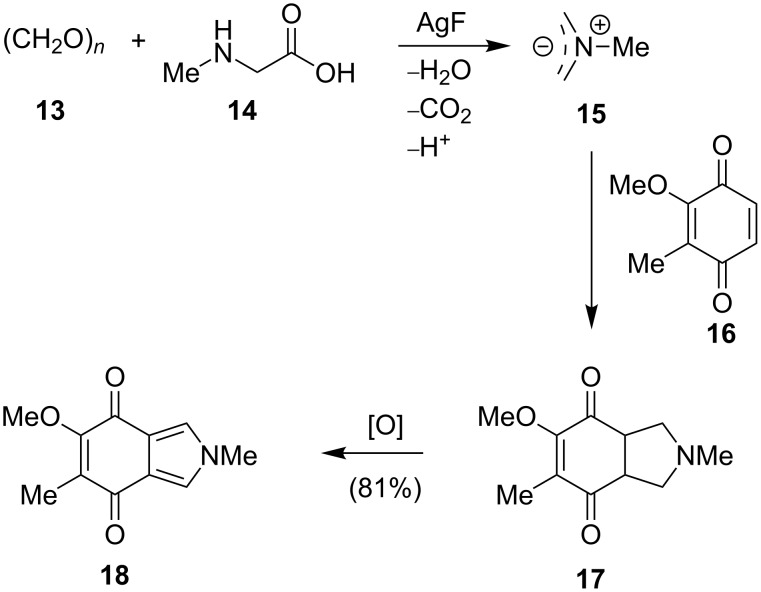
Synthesis of isoindole **18**.

The first naturally occurring isoindole, 6-methoxy-2,5-dimethyl-2*H*-isoindole-4,7-dione (**18**), was isolated from the sponge *Reniera* sp. in 1982 [10]. The postulated structure was elucidated through extensive NMR studies and unambiguously confirmed by a four-step synthesis. In 1991, a more concise and elegant route to this antimicrobial metabolite was established by Schubert-Zsilavecz [11]. The reaction was initiated by heating paraformaldehyde (**13**) and sarcosine (**14**) in the presence of benzoquinone **16**. This transformation proceeds via a 1,3-dipolar cycloaddition between the in situ formed azomethinylide **15** and the benzoquinone **16** to directly give **17** (Scheme 1). Spontaneous oxidation of the so-obtained cyclization adduct generates isoindole **18**.

**Scheme 2:**
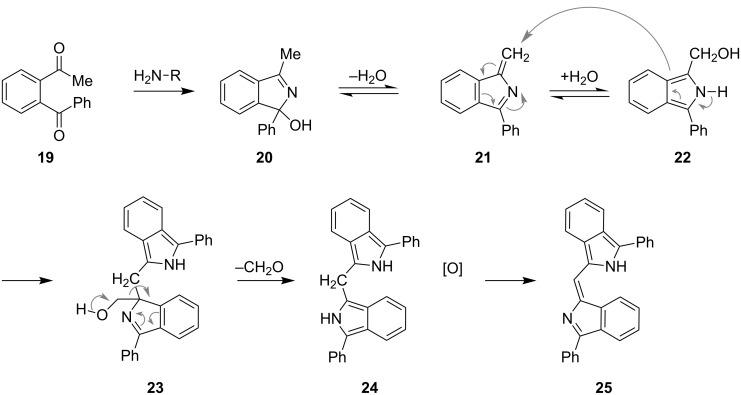
Staining amines with 1,4-diketone **19** (R = H).

Isoindoles have also found application as dyes. Pigment yellow 139 (**12**), which is sold by BASF as Paliotol^®^ Yellow K 1841 belongs to the class of highly resistant and effective 1,3-disubstituted isoindoline dyes. Recently, the use of isoindoles as red to near-infrared fluorophores was reported [12]. Another interesting isoindole-based dye, **25**, arises from the condensation of primary amines with *o*-diacylbenzene **19** (Scheme 2) [13]. After initial formation of **20**, isomerization to **21** and **22** can occur through a sequential dehydration–hydration process. Dimerization of **21** and **22** generates **23**, the substrate for a formal retro-Aldol reaction. Loss of formaldehyde gives **24**, which is spontaneously oxidized to the intensive blue-violet pigment **25**. This reaction sequence is characterized by its high sensitivity and has found application as a marker in analytical chemistry (e.g. staining of primary amines) [14–15].

The following review section highlights synthetic and biochemical aspects of selected isoindole-type natural products. The presented results are not meant to be exhaustive but should give a better understanding about the structurally and biologically most attracting structures. We intend to inspire today´s chemists and are convinced that these natural products have a huge potential for the development of new chemistry.

## Review

**Figure 2:**
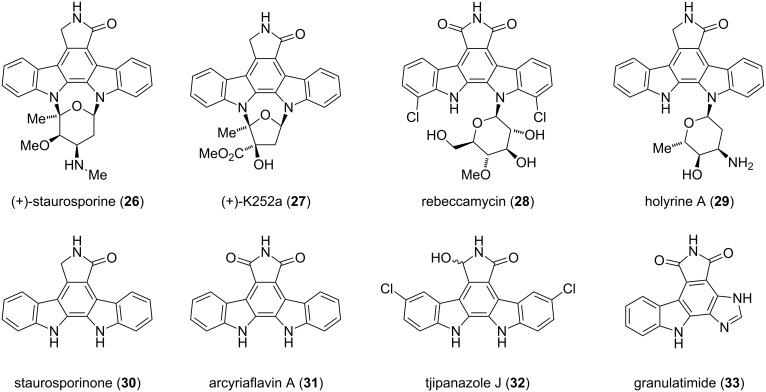
Representative members of the indolocarbazole alkaloid family.

**Indolocarbazoles.** Staurosporine (**26**) was the first member of the indolocarbazole alkaloid family to be discovered by Ōmura from *Streptomyces staurosporeus* at the Kitasato Institute in 1977 [16]. Over the past 35 years, more than 60 natural indolocarbazole compounds have been isolated from several bacteria and marine invertebrates either as their glycosides (**26**–**29**) or aglycones (**30**–**33**) [17]. Based on the number of glycosidic bonds linking the carbohydrate moiety to the isoindole framework, the latter can be divided into two subclasses. The first comprises two linkages, exemplified by (+)-staurosporine (**26**) and (+)-K252a (**27**), whereas the second class contains only one glycosidic bond as found in rebeccamycin (**28**) and holyrine A (**29**). Selected members of the indolocarbazole alkaloid family are depicted in Figure 2.

**Figure 3:**
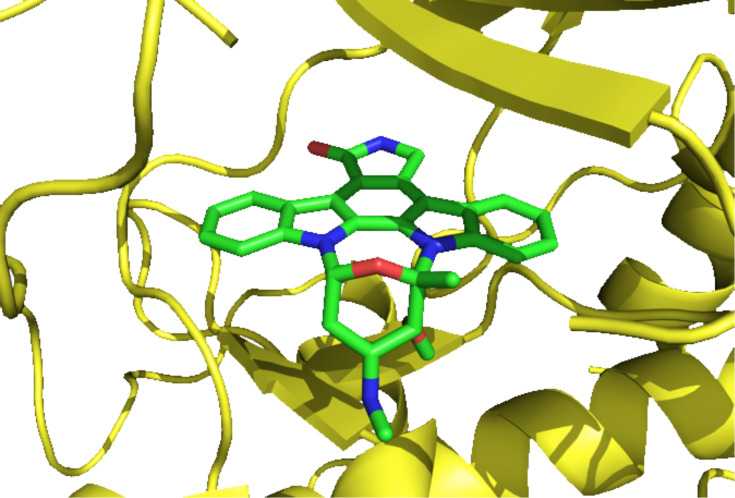
Staurosporine (**26**) bound to the adenosine-binding pocket [19] (from pdb1stc).

The strong antimicrobial activity against fungi and yeast of **26** was simultaneously reported with its isolation [16]. Moreover, with IC_50_ values in the low nanomolar range (1–20 nM), staurosporine (**26**) is one of the most potent protein kinase inhibitors to date [18]. It took nine years to identify the crucial protein kinase inhibitory effects of **26** [18]. Solving the crystal structure of the catalytic subunit Cα of cAMP-dependent protein kinase bound to **26** gave pivotal insights in the binding mode [19]. Staurosporine (**26**) targets the active site of the adenosine-binding pocket of most protein kinases. This is achieved mimicking several aspects of the adenosine moiety, by induced-fit structural changes and the conformational flexibility of the enzyme residues (Figure 3). Together with several related analogues, **26** displays significant cytotoxic and antiproliferative effects [19].

**Figure 4:**
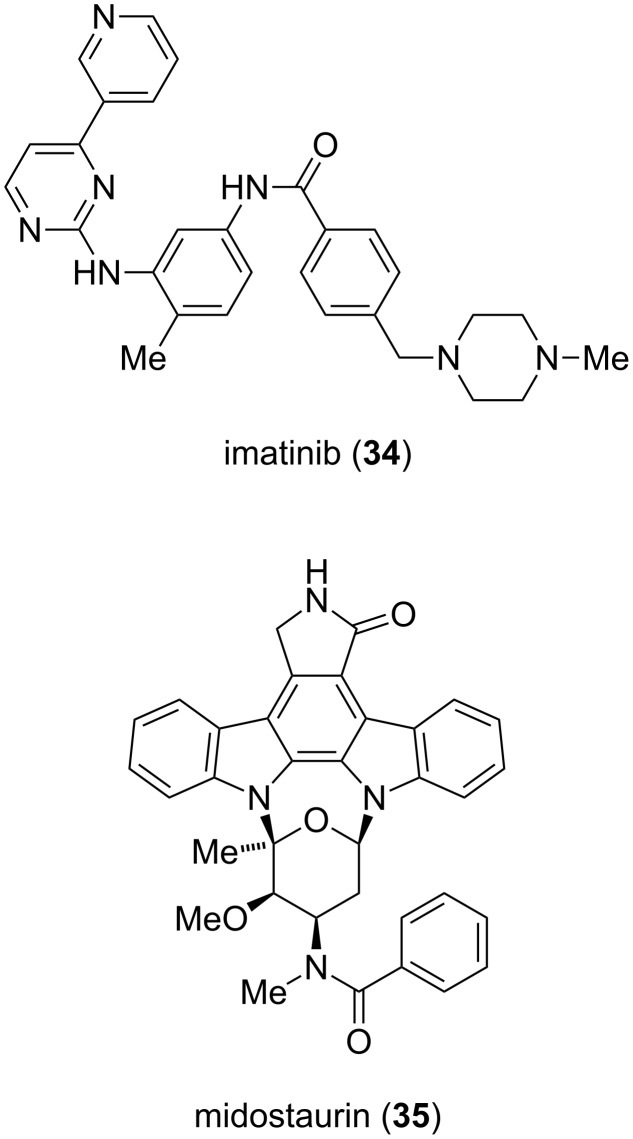
Structure of imatinib (**34**) and midostaurin (**35**).

Due to the key role of protein kinases in cellular signaling and their association with cancer, a tremendous effort in the development of selective protein kinase inhibitors was undertaken. This resulted in the discovery of the anticancer agent imatinib (Gleevec, **34**) by rational drug design (Figure 4). Midostaurin (**35**), a semisynthetic derivative of staurosporine (**26**), is currently in clinical trials for the treatment of acute myeloid leukemia [20].

Although rebeccamycin (**28**) and its congeners only differ in the lack of the second glycosidic linkage, a completely different mode of action is effective. By stabilizing the topoisomerase I DNA-cleavable complex they prevent the replication of DNA and act as efficient antitumor compounds [21].

**Scheme 3:**
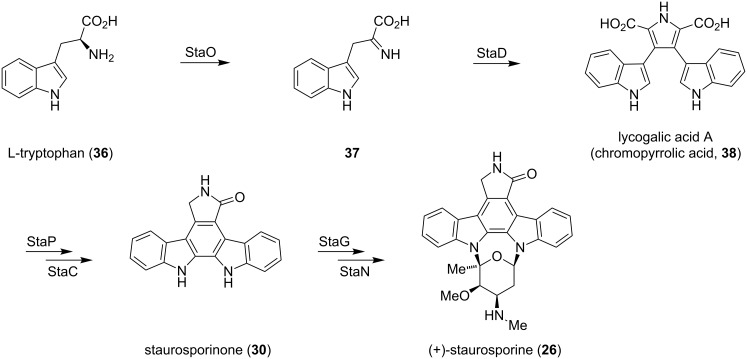
Biosynthesis of staurosporine (**26**).

The extraordinary bioactivity of indolocarbazoles has drawn a lot of attention from chemists and biologists. The biosynthesis of staurosporine (**26**) and rebeccamycin (**28**), both being the most prominent representatives of this distinct class of natural products, has been studied extensively and reviewed by Knölker and Ōmura [17,22]. The biogenesis of staurosporine (**26**) is outlined in Scheme 3. Biosynthetically, the indolocarbazole scaffold is derived from two fused tryptophan molecules, whereas the sugar moiety originates from glucose and methionine [23]. In 1996, Steglich isolated lycogalic acid A (**38**), also known as chromopyrolic acid, along with staurosporinone (**30**) and traces of arcyriaflavin A (**31**) from the extracts of *Lycogala epidendrum* [24]. This finding and results from Hoshino, which demonstrated that **38** is derived from L-tryptophan (**36**) [25], indicate a close biosynthetic relationship between these alkaloids. After additional experiments, the authors concluded that lycogalic acid A (**38**) is the biosynthetic key intermediate for the biogenesis of indolocarbazoles. This hypothesis was verified through gene disruption studies and the isolation of putative intermediates [26–28]. The formation of the isoindole framework is initiated by the oxidation of L-tryptophan (**36**) to imino indolepyruvic acid **37**, a process, which is catalyzed through the enzyme StaO. The subsequent condensation of two molecules of imine **37** is catalyzed by the oxidase StaD and gives lycogalic acid A (**38**) [26–28]. StaP and StaC convert **38** into staurosporinone (**30**) via an intramolecular aryl–aryl coupling and an oxidative decarboxylation [29–30]. Formation of the *N*-glycosidic bonds is carried out by StaG/StaN and after methylation, **26** is obtained [27].

**Scheme 4:**
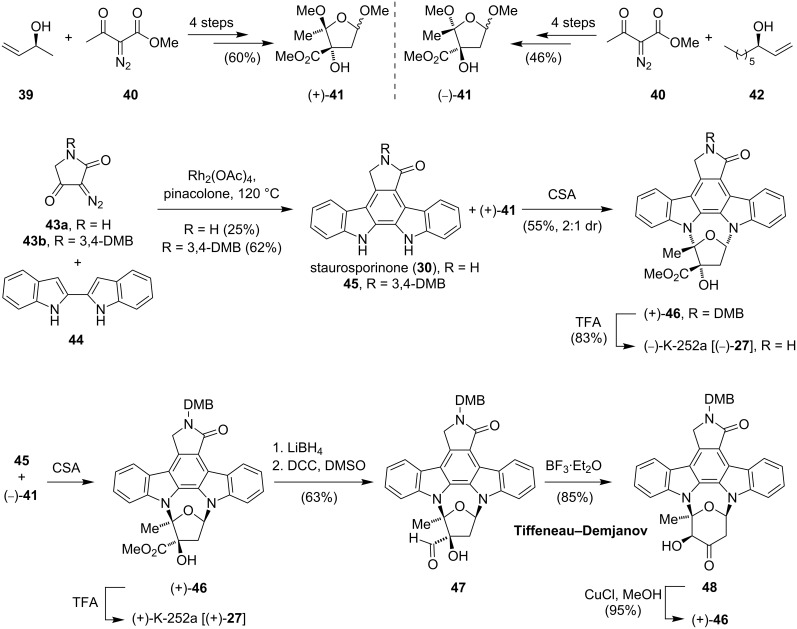
Wood’s synthesis of K-252a via the common intermediate **48**.

As already mentioned before, indolocarbazoles are characterized by their excellent biological activities and unusual architecture and hence drawing an enormous interest from the synthetic organic chemistry community. This led to several syntheses of the natural occurring aglycone staurosporinone (**30**) [31–35] and culminated in the first total synthesis of staurosporine (**26**) by Danishefsky in 1995 [36]. The elegant syntheses of a series of closely related indolocarbazoles, staurosporine (**26**), K252a (**27**), RK-286c (**49**), MLR-52 (**20**) and staurosporinone (**30**), which were elaborated by Wood in 1996 and reviewed by Knölker in more detail [37], will be discussed below. Based on their previous results [38], the common intermediate **48** was recognized as an ideal branching point. The synthetic route to this component is characterized by the beautiful application of Rh(II)-catalyzed C–H and OH insertion reactions (Scheme 4). The preparation of both enantiomeric furanose building blocks commenced with the Rh_2_(OAc)_4_-catalyzed OH insertion of **39**, respectively **40** into the α-diazo-β-ketoester **40**. A tandem [3,3]/[1,2]-rearrangement cascade, followed by reductive ozonolysis and acid-promoted cyclization afforded (+)-**41** and (−)-**41** in an overall yield of 60% and 46%, respectively. For the synthesis of staurosporinone (**30**) and its 3,4-dimethoxybenzyl (DMB)-protected derivative **45**, a ruthenium-catalyzed C–H insertion/electrocyclization cascade using 2,2’-bisindole **44** and diazolactams **43a/b** was developed. The necessary cycloglycosidative coupling of **45** and (+)-**41** was promoted by camphorsulfonic acid to give the DMB-protected K252a [(−)-**46**] as a 2:1 mixture, slightly favoring the desired diastereomer. Cleavage of the N-protecting group gave (−)-K252a [(−)-**27**] as its unnatural enantiomer. With eleven synthetic operations and a longest linear sequence of only seven steps, this route is highly convergent and employs a novel Rh-carbenoid-mediated formation of **30** as its key step. The synthesis of the natural enantiomer of (+)-K252a [(+)-**27**] was carried out in an analogous fashion using (*R*)-(−)-1-nonen-3-ol (**42**) instead of (*S*)-but-3-en-2-ol (**39**) to give the enantiomeric coupling partner of **45**. Although no yields for the following coupling were reported, multigram quantities of optically pure (+)-**46** could be obtained [37]. Further reduction and Moffat oxidation gave **47**, which was believed to be the direct precursor for the common intermediate **48**. Lewis acid catalyzed Tiffenau–Demjanov-type ring expansion of **47** gave **48** as a single product.

**Scheme 5:**
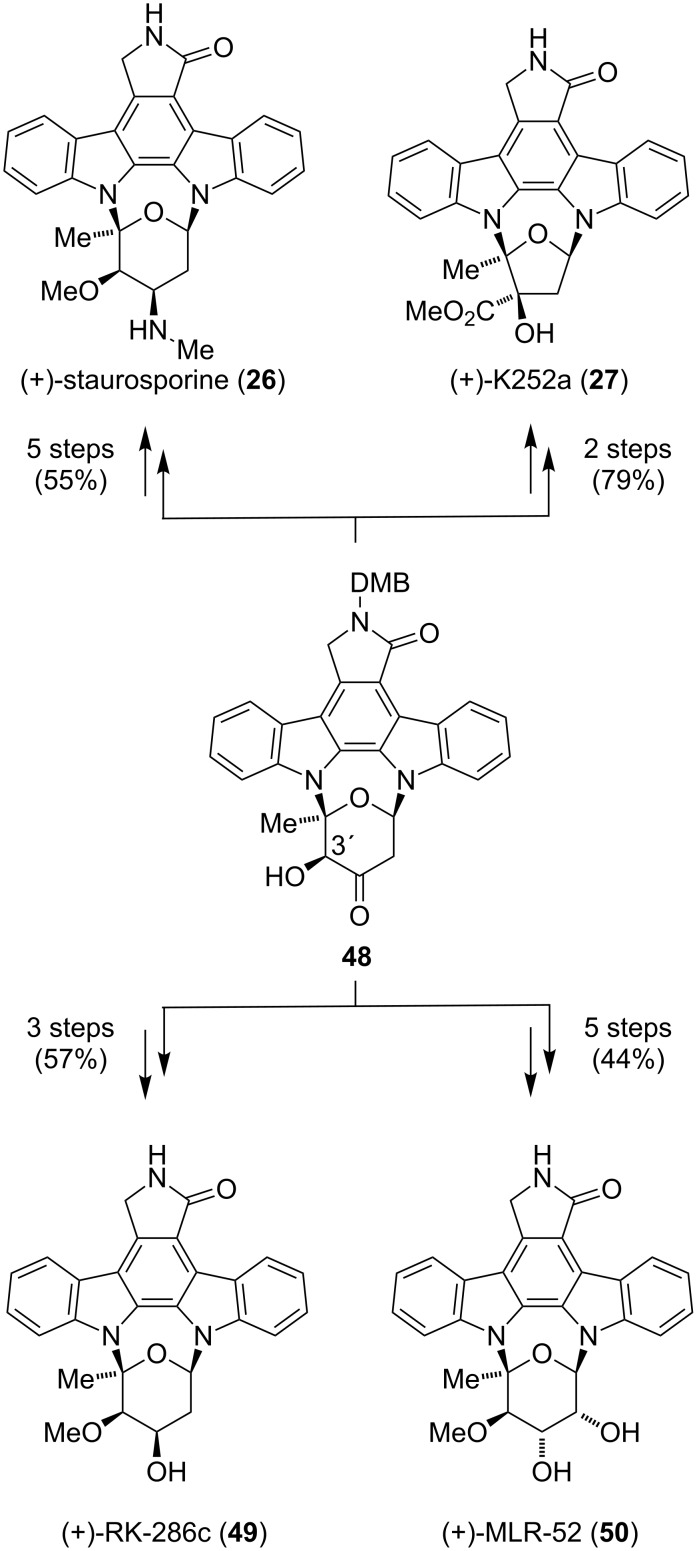
Synthesis of **26**, **27**, **49** and **50** diverging from the common intermediate **48**.

With α-hydroxy ketone **48** in hand, the stage was set for further modifications to access **26**, **27**, **49** and **50** (Scheme 5). During their attempts to methylate **48**, an unprecedented benzilic acid-type rearrangement (ring contraction) was discovered. Exposure of **48** to copper(I) chloride in methanol furnished (+)-**46** in 95% yield, which after removal of the protecting group gave (+)-K252a (**27**). Staurosporine (**26**) could be synthesized in five steps from **48** and in a longest linear sequence of 19 steps. For the preparation of (+)-RK-286c (**49**) and (+)-MLR-52 (**50**), ketone **48** was reduced and regioselectively methylated at the C3’ position. Cleavage of the protecting group gave **49**, whereas **50** was obtained after dehydration using Martin’s sulfurane, dihydroxylation and a final deprotection step.

**Figure 5:**
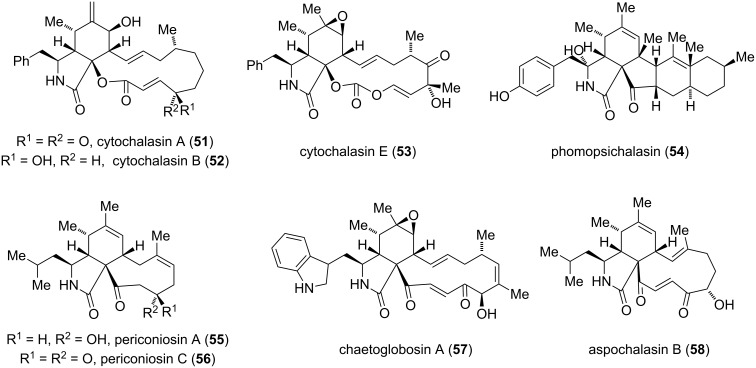
Selected members of the cytochalasan alkaloid family.

**Macrocyclic polyketides:** The first cytochalasan alkaloids, cytochalasin A (**51**) and B (**52**), originally named phomins [39], were isolated in 1966 [39–42]. Since then, the number of natural products belonging to this family has increased to over 80. Some representative members are depicted in Figure 5 [43]. Cyctochalasan alkaloids display a wide range of biological properties, such as cytotoxic, antimicrobial, antiviral and phytotoxic activities and were reviewed in detail by Hertweck in 2010 [44]. Structurally, they consist of one amino acid and a highly substituted hydroisoindolone moiety fused to a macrocyclic ring (ring size 9–14). In certain cases, the ring system can be oxidized to an unusual cyclic carbonate as observed for cytochalasin E (**53**). Depending on the amino acid incorporated in the isoindolone moiety, the cytochalasans can be further subdivided into cytochalasins [41] (phenylalanine), chaetoglobosins [45–46] (tryptophan), aspochalasins [47] (leucine), pyrichalasins [48] (tyrosine) or alachalasins [49–50] (alanine).

**Scheme 6:**
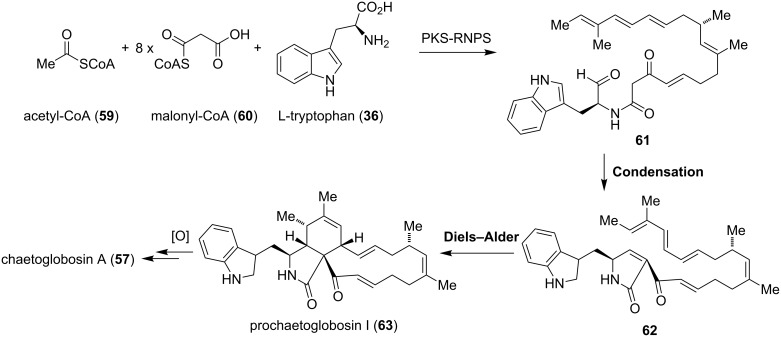
Biosynthesis of chaetoglobosin A (**57**) [56].

The biosynthesis of cytochalasans was established on the basis of various isotope labeling experiments using cytochalasin B (**52**) as a model system [51–55]. It was hypothesized that the carbon backbone, which is connected to an amino acid, most likely originates from a polyketide synthase (PKS)/nonribosomal peptide synthetase (NRPS) hybrid machinery [56]. The discovery of a gene locus for a PKS-NRPS synthetase of the chaetoglobosin producing fungus *Penicillium expansum* led to a more sophisticated biosynthetic insight of chaetoglobosin A (**57**) (Scheme 6). The stepwise assembly of the nonaketide **61** is realized from activated tryptophan (**36**), one acetyl-CoA (**59**) starter and eight malonyl-CoA extender (**60**) units. The remaining methyl groups are believed to be installed by *S*-adenosyl methionine (SAM). An intramolecular Aldol condensation generates the pyrrolinone **62**, which reacts via an [4 + 2]-cycloaddition to prochaetoglobosin I (**63**) [56]. Recently, gene disruption studies of *Chaetomium globosum* revealed the enzymes involved in the final oxidative transformations leading to chaetoglobosin A (**57**) [57].

**Scheme 7:**
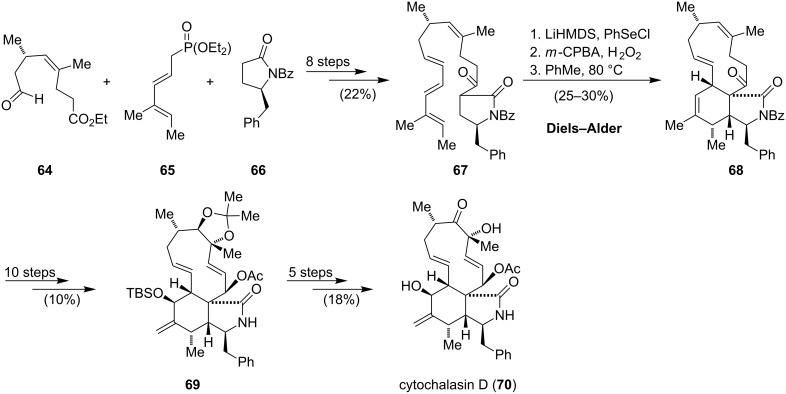
Synthesis of cytochalasin D (**70**) by Thomas [63].

The diverse biological activities and unique molecular architecture of the cytochalasan alkaloids have made them a famous synthetic target. Total syntheses of several members of the cytochalasans were reported, which were reviewed by Hertweck and Bräse [44,58]. In line with the focus on the construction of the isoindole component, one can identify the Diels–Alder reaction as the most popular strategy. A linear, biomimetic synthesis using a late stage intramolecular Diels–Alder reaction was implemented in the Stork synthesis of cytochalasin B (**52**) [59] and the Thomas synthesis of cytochalasin H, D, G and O [60–63]. An intermolecular reaction was reported for the synthesis of aspochalasin B (**58**) by Trost and Vedejs [64–67]. The synthesis of cytochalasin D (**70**) by Thomas is outlined in Scheme 7. The intermediate **67**, which contains all of the carbon atoms of the natural product, was synthesized in eight steps starting from the advanced building blocks **64**, **65** and **66**. Selective deprotonation and trapping the so-formed enolate with phenylselenyl chloride gave an intermediate selenide. Oxidation and elimination of the corresponding selenoxide at elevated temperature generated the necessary dienophile, which directly underwent an intramolecular *endo*-Diels–Alder reaction to give the isoindolinone **68**. For the synthesis of **69** another ten steps were necessary to discriminate between the three remaining double bonds, functionalize the full carbon skeleton and set the remaining stereocenters. Five steps, including the exchange of protecting groups and one oxidation, completed the synthesis of cytochalasin D (**70**).

**Scheme 8:**
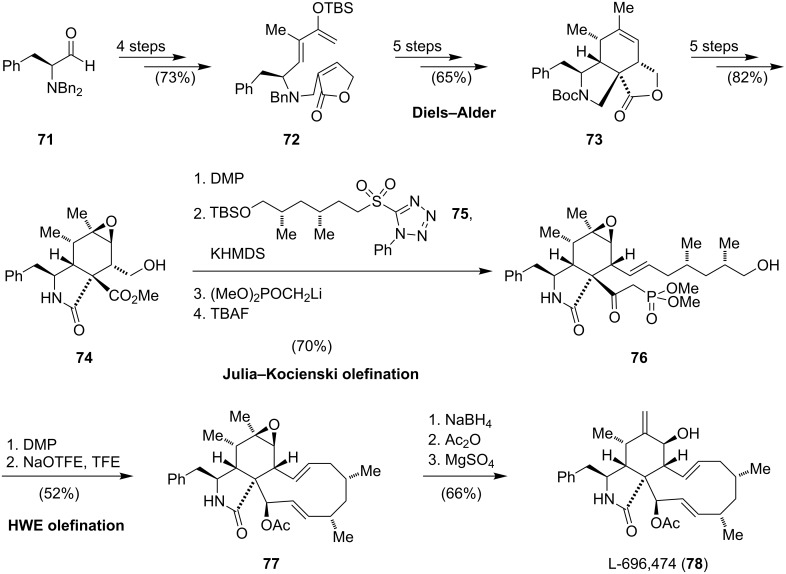
Synthesis of L-696,474 (**78**).

In comparison to the syntheses mentioned before (Stork, Thomas, Trost, Vedejs), the convergent approach reported by Myers allows a modular entry to diverse members of the cytochalasin alkaloid family [68]. As proof of concept, the macrolactone cytochalasin B (**52**) and the carbocyclic cytochalasin L-696,474 (**78**), a potent HIV-1 protease inhibitor [69–72], were synthesized (Scheme 8). Strategic bond disconnections revealed the common isoindolinone precursor **73**. The synthesis of the latter commenced from *N*,*N*-dibenzylphenylalanine (**71**) to afford the Diels–Alder substrate **72** in four steps. The envisioned intramolecular Diels–Alder cyclization of the silyl enol ether **72** provided the depicted *endo*-diastereomer in good yield. Exchange of the *N*-benzyl for a Boc-protecting group and cleavage of the silyl enol ether gave the corresponding ketone, which was first converted to an enol-triflate and then to the tricyclic alkene **73**. At this stage, the syntheses of cytochalasin B (**52**) and cytochalasin L-696,474 (**78**) diverged. For **78**, another five steps were necessary to form the isoindolinone **74**. After oxidation of the primary alcohol with Dess–Martin periodinane, the obtained aldehyde was coupled with the readily available *N*-phenyltetrazole sulfone **75** via a Julia–Kocienski olefination. Installation of the phosphonate and desilylation gave **76**, which, after oxidation, reacted in the presence of sodium 2,2,2-trifluoroethanol (NaOTFE) in 2,2,2-trifluoroethanol (TFE) via an intramolecular Horner–Wadsworth–Emmons reaction to **77**. Cytochalasin L-696,474 (**78**) was obtained from **77** via reduction, acetylation and a magnesium sulfate induced rearrangement of the epoxide to the allylic alcohol. The synthesis of cytochalasin B **52** (15 steps from **73**) proceeded in a similar fashion and was highlighted in detail by Hertweck [44]. The authors state that this approach is highly diversifiable and allows for a rapid access to a variety of members of the cytochalasin alkaloid family.

**Scheme 9:**
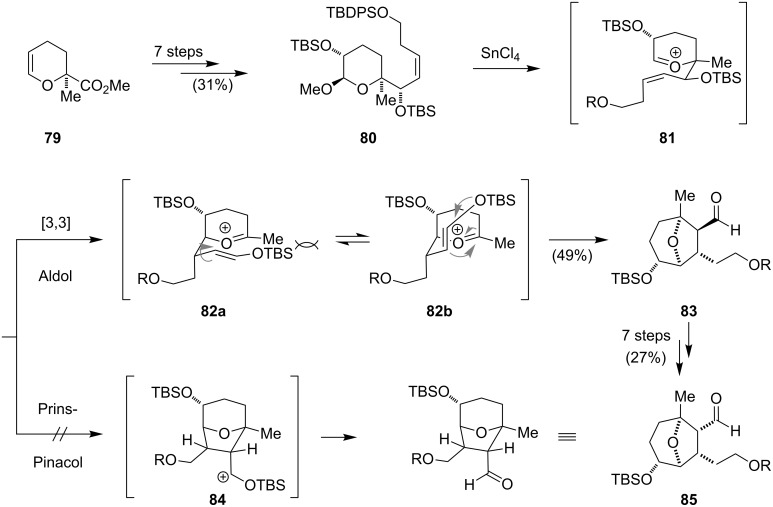
Synthesis of aldehyde **85** (R = TBDPS).

The power of the intramolecular Diels–Alder strategy for the synthesis of the isoindolinone moiety was also recognized during the synthesis of aspergillin PZ (**91**) [73]. This secondary metabolite has a remarkable architecture and was first isolated from the fungus *Aspergillus awamori* in 2002 [74]. The highly functionalized pentacyclic skeleton comprises a unique 12-oxatricyclo[6.3.1.0^2,7^]dodecane ring system and ten stereogenic centers. Its carbon framework is closely related to other members of the aspochalasin/cytochalasin alkaloids such as cytochalasin D (**70**), which lacks the unusual oxatricyclo ring system. The novel structure and cytotoxic activities of natural analogues of aspergillin PZ (**91**) against the HL-60 cancer cell line (IC_50_ = 50–80 nM) initiated Tanis’ synthetic program to further evaluate the therapeutic potential of **91** and derivatives thereof (Scheme 9) [73]. The synthesis could be accomplished in 26 steps and includes two key steps: the 8-oxabicyclo[3.2.1]octane unit was synthesized via an unprecedented 2-oxonia-[3,3]-sigmatropic aldol reaction cascade and the isoindolinone moiety was assembled using an intramolecular Diels–Alder reaction. The synthetic endeavor began with the conversion of dihydropyran **79** to the acetal **80** (Scheme 9). Activation of the anomeric position with tin(IV) chloride presumably led to the formation of the oxonium ion **81**. This intermediate was envisioned to undergo a Prins–Pinacol rearrangement via intermediate **84** to give the *cis*-aldehyde **85**. However, only the diastereomeric *trans*-aldehyde **83** was isolated in moderate yields from the reaction mixture. The authors concluded that a competing 2-oxonia-[3,3]-sigmatropic aldol pathway via **82a** and **82b** might be operative for this system. The stereochemical outcome could be attributed to the sterically less demanding transition state **82b**. The epimerization of **83** to **85** proceeded via an intermediate lactone and extended the route by seven steps.

**Scheme 10:**
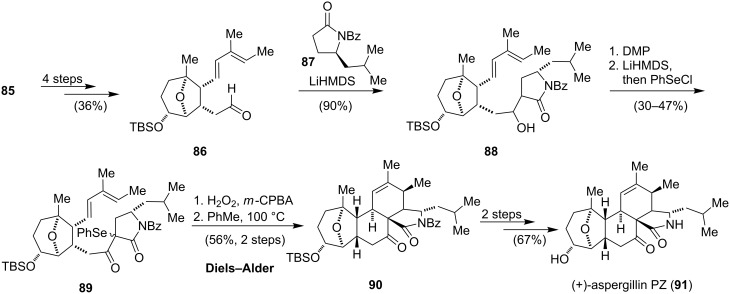
Synthesis of (+)-aspergillin PZ (**79**) by Tanis.

After having secured the *cis*-epimer **85**, the diene moiety was introduced in four steps to give aldehyde **86**, which was reacted with the lithium enolate of lactam **87** to furnish the aldol adduct **88** (Scheme 10). Oxidation of the secondary alcohol to the corresponding ketone and selective selenation with phenylselenyl chloride gave **89**. Following the reported sequence for the synthesis of cytochalasin D (**70**), namely oxidation of the selenide to the selenoxide, elimination and intramolecular Diels–Alder reaction, isoindolinone **90** could be obtained in moderate yield. Removal of the protecting groups then yielded aspergillin PZ (**91**).

Though having established a rather long and low yielding synthesis, ample quantities could be obtained for a preliminary evaluation of the biological activity of **79**. In a first screen against A2058 melanoma and DU145 prostate cancer cell lines, **79** showed to be inactive [73].

**Figure 6:**
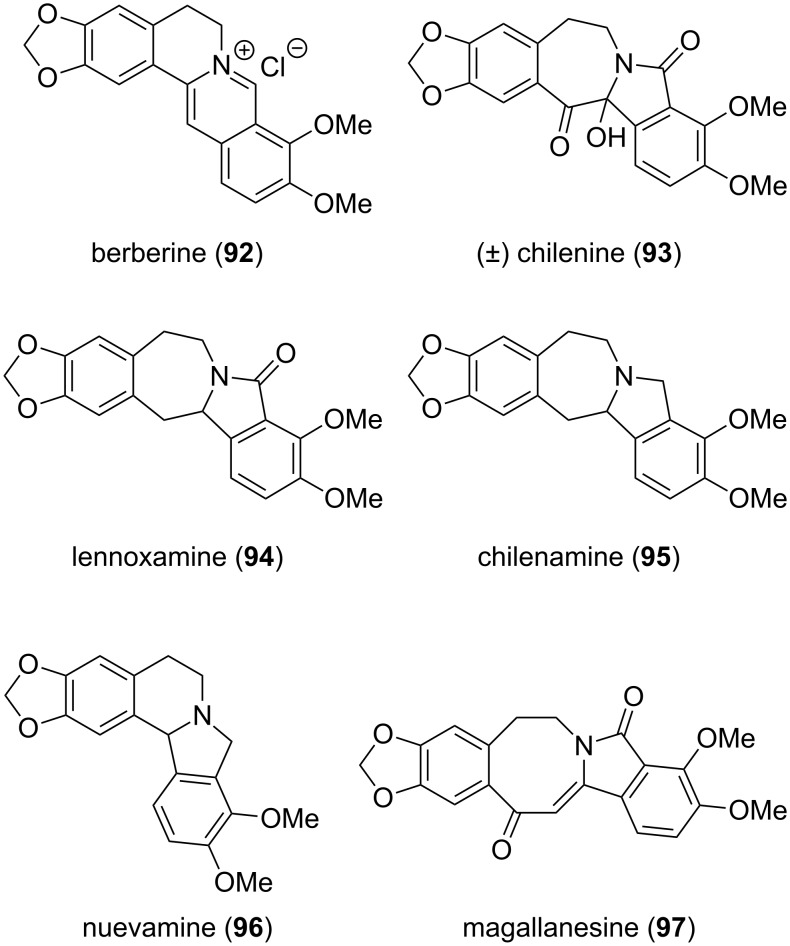
Representative *Berberis* alkaloids.

**Isoindolinones derived from isoquinoline alkaloids:** The aporhoeadane alkaloids, a term designated by Shamma [75], are abundantly found in South American members of the botanical family of *Berberis* (*Berberidaceae*). Along with the ever present isoquinoline berberine (**92**), the isoindole-containing benzazepines chilenine (**93**) [76], lennoxamine (**94**) [77] and chilenamine (**95**) [78], the isoquinoline nuevamine (**96**) [77] and the benzazocine magallanesine (**97**) [79] were isolated from different *Berberidaceae* species (Figure 6).

**Scheme 11:**
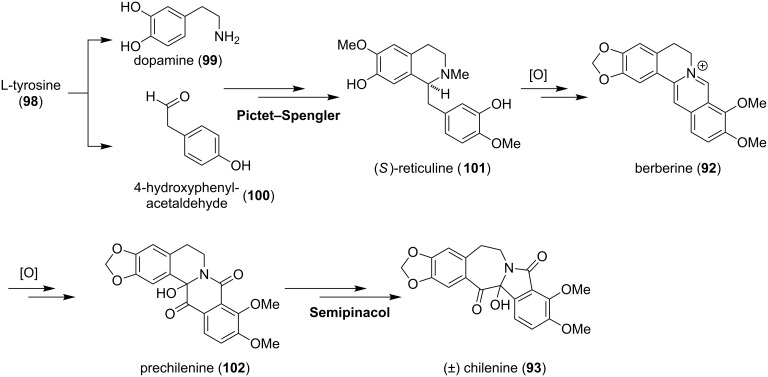
Proposed biosynthetic pathway to chilenine (**93**).

Chilenine (**93**) was the first isoindolobenzazepine alkaloid isolated from *Berberis empetrifolia* in 1982. The biogenesis proceeds most likely via a Pictet–Spengler reaction of dopamine (**99**) with 4-hydroxyphenylacetaldehyde (**100**), both derived from L-tyrosine (**98**) (Scheme 11). After oxidation and *O*-methylation, which is carried out by *S*-adenosylmethionine (SAM), (*S*)-reticuline (**101**) is obtained. Oxidation of the *N*-methyl group to the iminium ion and cyclization gives the tetracyclic carbon skeleton, which upon further methylation and oxidation furnishes berberine (**92**) [80]. Prechilenine (**102**), itself isolated as its *O*-methyl ether is formed via oxidation [78]. A base-catalyzed semipinacol-type rearrangement yields **93** [76]. Chilenine (**93**) can be further transformed to the [4.4.0]-tricycle lennoxamine (**94**) and the [5.3.0]-ring system chilenamine (**95**) [77–79]. Other members such as nuevamine (**96**) and magallanesine (**97**) contain the corresponding [4.3.0] and [6.3.0]-scaffold.

Since their first isolation in the 80’s of the last century, a myriad of syntheses of these small, sometimes highly functionalized and mainly biological inactive alkaloids have been described in the literature. Most of this work is summarized in an excellent review by Leonard [81]. We confine this section to the syntheses of magallanesine (**97**) by Danishefsky and Kurihara, which were also mentioned by Evans and Bentley [82–83].

**Scheme 12:**
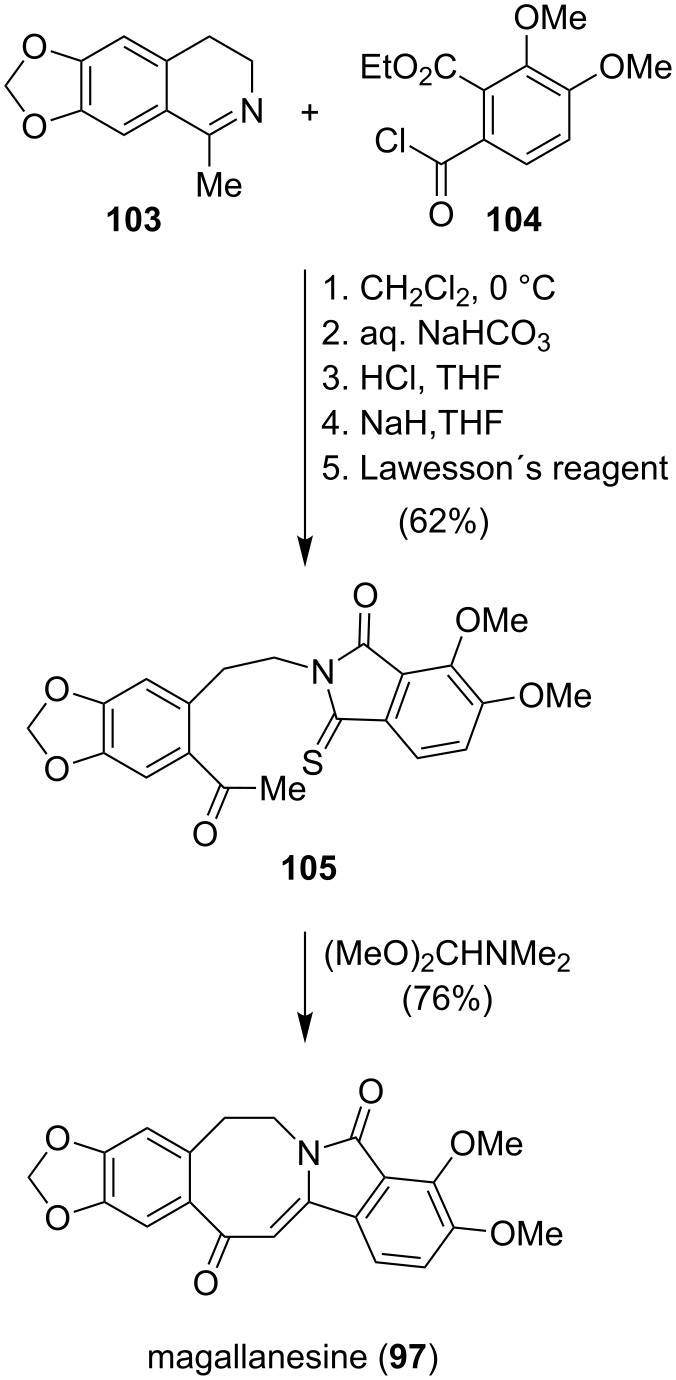
Synthesis of magallanesine (**97**) by Danishefsky [84].

Magallanesine (**97**), which was isolated in 1985 from *Berberis darwinii*, a plant native to southern Chile and Argentina, is the first known isoindolobenzazocine alkaloid. The seminal total synthesis of **97** was achieved in the group of Danishefsky (Scheme 12) using a dimethylformamide acetal-mediated cyclodehydration of **105**. The isoindole unit of **105** was prepared from the condensation of **103** and **104**. Oxygen–sulfur exchange provided the thiophthalimide **105**, which could be converted to magallanesine (**97**) via an intramolecular aldol condensation in one step [84].

**Scheme 13:**
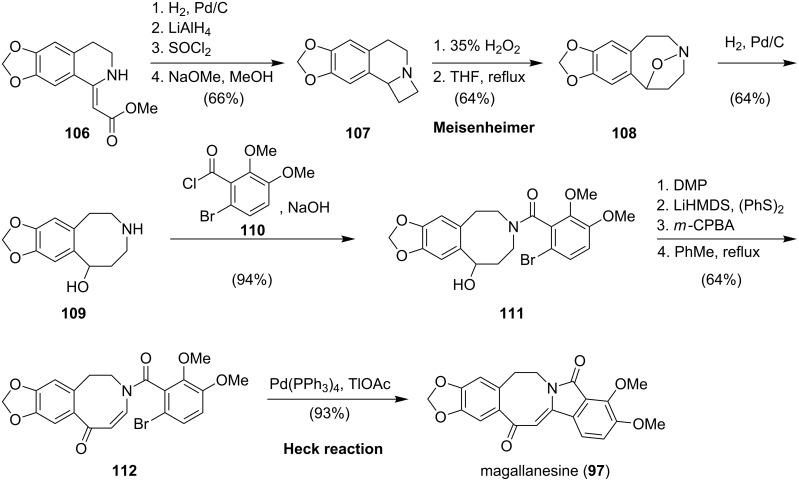
Kurihara’s synthesis of magallanesine (**85**).

In 1996, Kurihara (Scheme 13) reported a [1,2]-Meisenheimer rearrangement followed by an intramolecular Heck cyclization to elaborate the isoindolobenzazocine moiety [85]. The synthesis commenced with the preparation of ester **106** from piperonal according to a known procedure [86]. A four-step sequence yielded azetidine **107**. After oxidation with hydrogen peroxide, the resulting *N*-oxide cleanly underwent a [1,2]-Meisenheimer rearrangement upon heating in tetrahydrofuran. The so-formed azocine **108** was converted to amine **109** by hydrogenolysis of the N–O bond. Amide formation with acid chloride **110** gave **111**, which upon sequential oxidation yielded enaminone **112**. Finally, the isoindolinone moiety was generated via an intramolecular Heck reaction using tetrakis(triphenylphosphine)palladium(0) and thallium acetate, to give magallanesine (**97**) in excellent yield [85].

**Aporphine alkaloids:** The aristolactam alkaloids are classified as members of the aporphine alkaloid family and contain a characteristic phenanthrene lactam core. Aristolactams (**113**–**116**) along with aristolochic acids (**117**,**118**) and 4,5-dioxoaporphines (**119**,**120**) were mainly isolated from *Aristolochiaceae* but can also be found in several other plant species (Table 1) [87–92]. Several comprehensive overviews of various aristolactams were published by Shamma, Parmar and Bentley [87,92–95].

**Table 1:** Selected members of the aporphine alkaloid family.

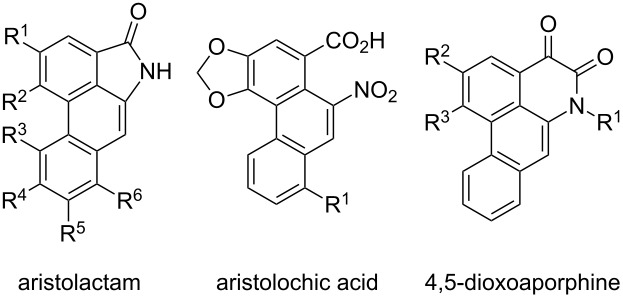						

	R^1^	R^2^	R^3^	R^4^	R^5^	R^6^

**aristolactams**						
aristolactam I (**113**)	O–CH_2_–O		H	H	H	OMe
aristolactam AII (**114**)	OH	OMe	H	H	H	H
aristolactam BII (**115**)	OMe	OMe	H	H	H	H
enterocarpam II (**116**)	OH	OMe	H	H	H	OMe
**aristolochic acids**						
aristolochic acid I (**117**)	OMe					
aristolochic acid II (**118**)	H					
**4,5-dioxoaporphines**						
aristolodione (**119**)	Me	OH	OMe			
cepharadione A (**120**)	Me	O–CH_2_–O				

**Scheme 14:**
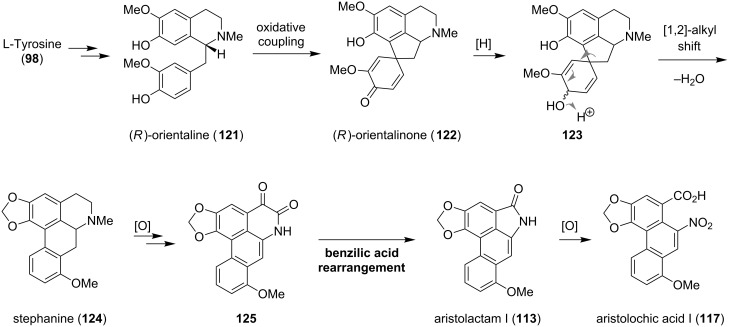
Proposed biosynthesis of **113**, **117** and **125**.

The co-occurrence of aristolactams, aristolochic acids and 4,5-dioxoaporphines led to the assumption of a close biogenetic relationship between these three classes [87,96]. The biosynthesis of aristolochic acid I (**117**) was elucidated via labeling experiments and is depicted in Scheme 14 [97–100]. First, two molecules of the amino acid L-tyrosine (**98**) are transformed to (*R*)-orientaline (**121**) in a similar fashion as described for the biogenesis of (*S*)-reticuline (**101**) (compare Scheme 11). A de-aromatizing spirocyclization of **121** leads to (*R*)-orientalinone (**122**), which, after reduction of the ketone to the secondary alcohol **123**, undergoes a [1,2]-alkyl migration with concomitant loss of water. Re-aromatization and dioxolane formation gives stephanine (**124**). It is hypothesized that direct oxidation of **124** forms 4,5-dioxoaporphine **125**, which upon benzilic acid rearrangement, *N*-methyl cleavage and decarboxylation provides aristolactam I (**113**) [96]. Additional oxidation of the amide function furnishes aristolochic acid I (**117**).

**Scheme 15:**
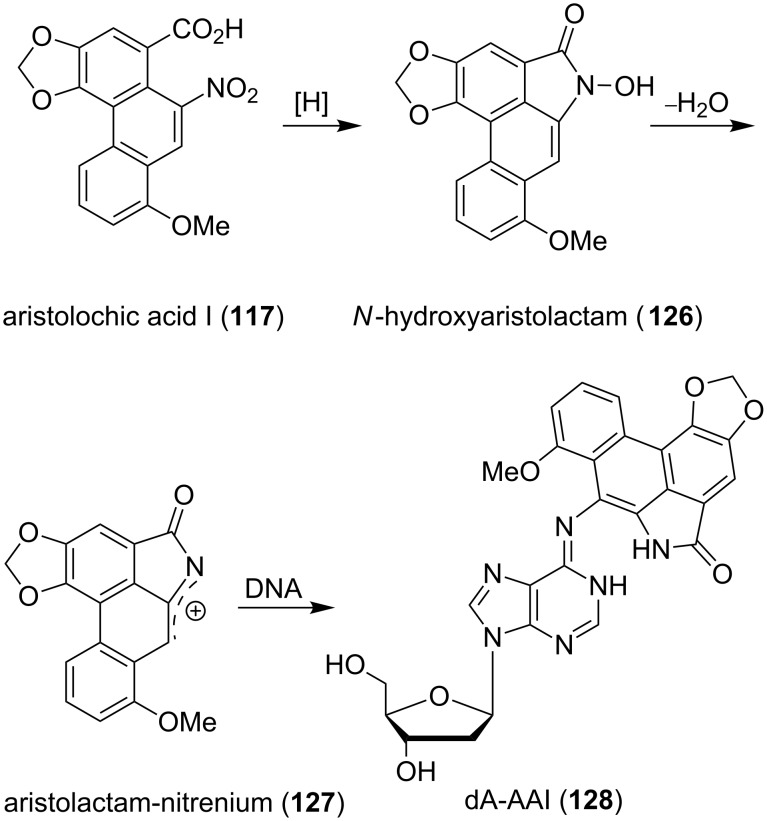
DNA lesion caused by aristolochic acid I (**117**) [102].

The aristolactams and aristolochic acids have a broad spectrum of biologically interesting properties. Although both can be isolated from the same plants, their biological activity is highly variable. Herbal drugs containing aristolochic acids display cytotoxic activities and inhibitory effects on platelet aggregation and were traditionally used in folk medicine [101]. However, since the early 1980’s aristolochic acids are associated with the development of several cancer types and nephropathy (Chinese herbs nephropathy). The mechanism for the formation of covalent DNA adducts is shown in Scheme 15 [102–103]. The DNA damage is initiated by metabolic reduction and cyclization of **117** to *N*-hydroxyaristolactam **126**. After loss of water, the aristolactam nitrenium ion **127**, which is believed to be the main carcinogen, reacts with DNA bases such as adenosine, to give the corresponding 7-(deoxyadenosin-*N*^6^-yl)aristolactam I (dA–AAI, **128**).

Interestingly, aristolactams are not mutagenic themselves but show immunosuppressant [104], antiplatelet [105], antimycobacterial [106], neuroprotective [107] activities and are excellent inhibitors of cyclin-dependent kinases (CDKs) with IC_50_ values in the nanomolar range [108–109]. CDKs are regulatory proteins, which play an important role in the cell cycle and are considered to be a potential target for anticancer treatment. This makes the aristolactams promising lead components for further drug discovery [108]. The similarity of the aromatic moiety of aristolactams and staurosporines, which are also known to be potent kinase inhibitors could explain the observed inhibition of cyclin-dependent kinases [109]. Over the past years, considerable synthetic efforts have been dedicated to the aristolactams and the development of non-natural analogues.

**Scheme 16:**
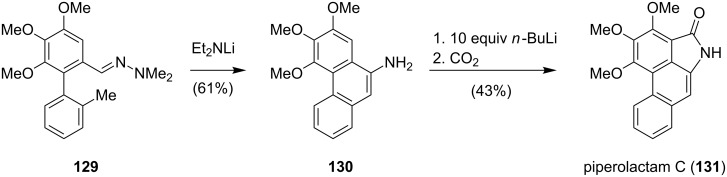
Snieckus’ synthesis of piperolactam C (**131**).

Pioneering work was carried out in the groups of Castedo [110] and Couture [111–112]. A beautiful application of a directed *ortho*-metalation (DoM) strategy was reported in the synthesis of piperolactam C (**131**) by Snieckus (Scheme 16) [113]. Treatment of **129** with base afforded aminophenanthrene **130** via a remote lateral metalation–cyclization sequence. Metalation of **130** with excess *n*-butyllithium followed by carbonation then yielded piperolactam C (**131**).

**Scheme 17:**
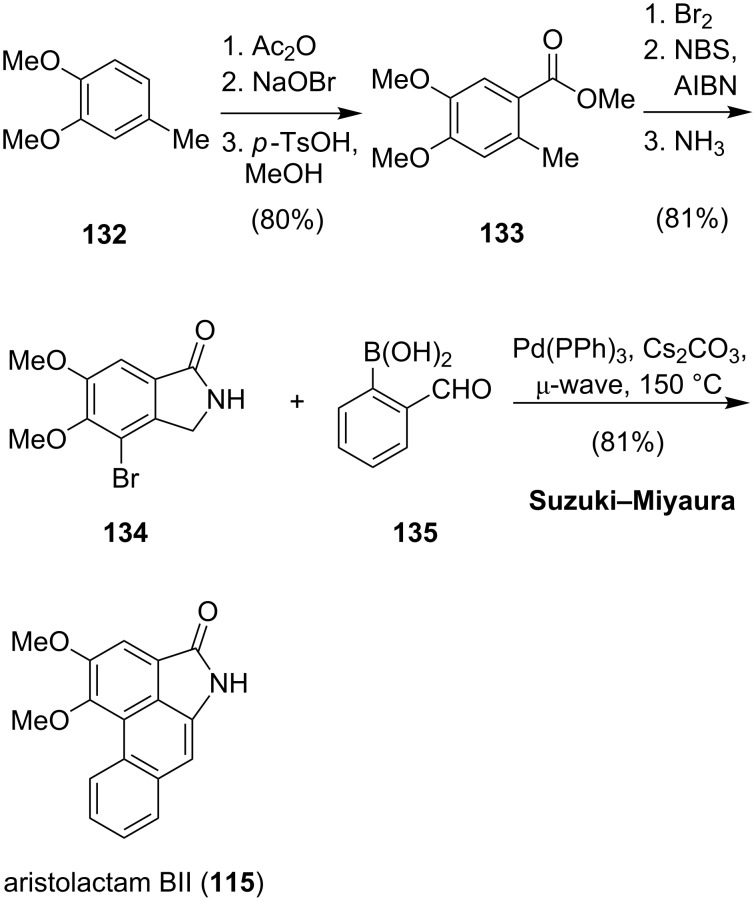
Synthesis of aristolactam BII (**104**).

In 2008, Heo and coworkers reported the synthesis of several aristolactams employing a one-pot cross-coupling/aldol condensation cascade reaction (Scheme 17) [91]. The synthesis commenced with the preparation of the isoindolinone building block **134**. Friedel–Crafts acetylation of **132**, followed by a Lieben haloform degradation gave the corresponding acid, which after methylation yielded **133**. Bromination and lactam formation afforded isoindolinone **134**. The following one-pot Suzuki–Miyaura/aldol condensation of **134** with boronic acid **135** was carried out at 150 °C in a microwave reactor and gave aristolactam BII (**115**) in good yield. By variation of the coupling partners, Heo was able to efficiently synthesize a library of natural and non-natural analogues.

**Figure 7:**
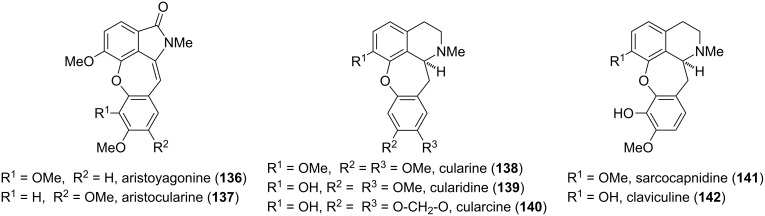
Representative cularine alkaloids.

**Cularine alkaloids:** Another prominent class of isoquinoline alkaloids, typically found in the botanical family of *Fumariaceae* and in particular in *Sarcocapnos enneaphylla*, are the cularines (**136**–**142**) (Figure 7) [114–116]. In comparison to the related aporphine alkaloids, the cularine skeleton contains a benzoxepine ring system. The parent alkaloid (+)-cularine (**138**) was first isolated by Manske in 1938 [117], and its molecular skeleton was assigned on the basis of NMR and X-ray crystallographic studies. The structure elucidation revealed a twist-boat conformation of the dihydrooxepine ring [118–120]. Within this family, aristoyagonine (**136**), which was first isolated by Castedo in 1984, is the only known naturally occurring member of the cularine alkaloids bearing an isoindolinone moiety [121]. Aristocularine (**137**), a synthetic derivative of aristoyagonine (**136**), is known to display cytotoxic activities in the micromolar range [122].

**Scheme 18:**
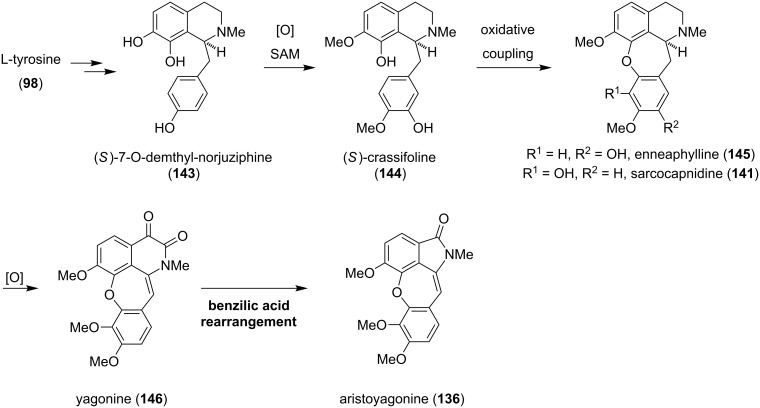
Proposed biosynthesis of **136**.

Biosynthetically, the cularines can be traced back to L-tyrosine (**98**) (Scheme 18) [123–124]. Incorporation of radioactively labeled L-tyrosine into crassifoline (**144**), which is an established cularine precursor, most likely occurs via alkaloid **143**. Hydroxylation and sequential methylation of **143** leads to **144**. Oxidative phenol coupling then forms the cularine alkaloid skeleton. The generated cularine enneaphylline (**145**) and the regioisomeric *iso*-cularine sarcocapnidine (**141**) could serve as branching points for the synthesis of further cularine derivatives. Sequential oxidations of **141**, similar to the biosynthesis of the aristolactams (Scheme 14), yields yagonine (**146**), which could be converted to aristoyagonine (**136**) via a benzilic acid-type rearrangement [121].

**Scheme 19:**
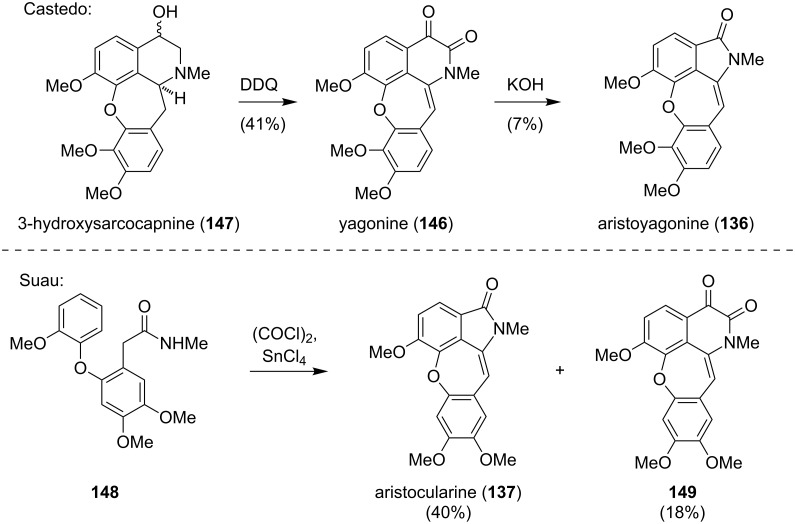
The syntheses of **136** and **137** reported by Castedo and Suau.

The first syntheses of aristoyagonine (**136**) were reported by Castedo and Suau (Scheme 19) [121–122,125–127]. The approach carried out by Castedo relied on 3-hydroxysarcocapnine (**147**), which could either be isolated from natural sources or prepared via an Ullmann coupling of a 1-(2-bromobenzyl)-8-hydroxyisoquinoline derivative [121,125]. Oxidation of **147** with 2,3-dichloro-5,6-dicyano-1,4-benzoquinone (DDQ) gave the dioxocularine yagonine (**146**). The bioinspired, base-mediated transformation of **146** to **136** obviously proceeds via a benzilic acid rearrangement–oxidation sequence.

In 1996, the group of Suau accessed aristocularine **137** along with **149** in one step from acetamide **148**. In the presence of oxalyl chloride and the Lewis acid tin(IV) chloride, a tandem cyclization (Bischler–Napieralski/Friedel–Crafts acylation reaction) was triggered to directly give **137** and **149** [122,127].

**Scheme 20:**
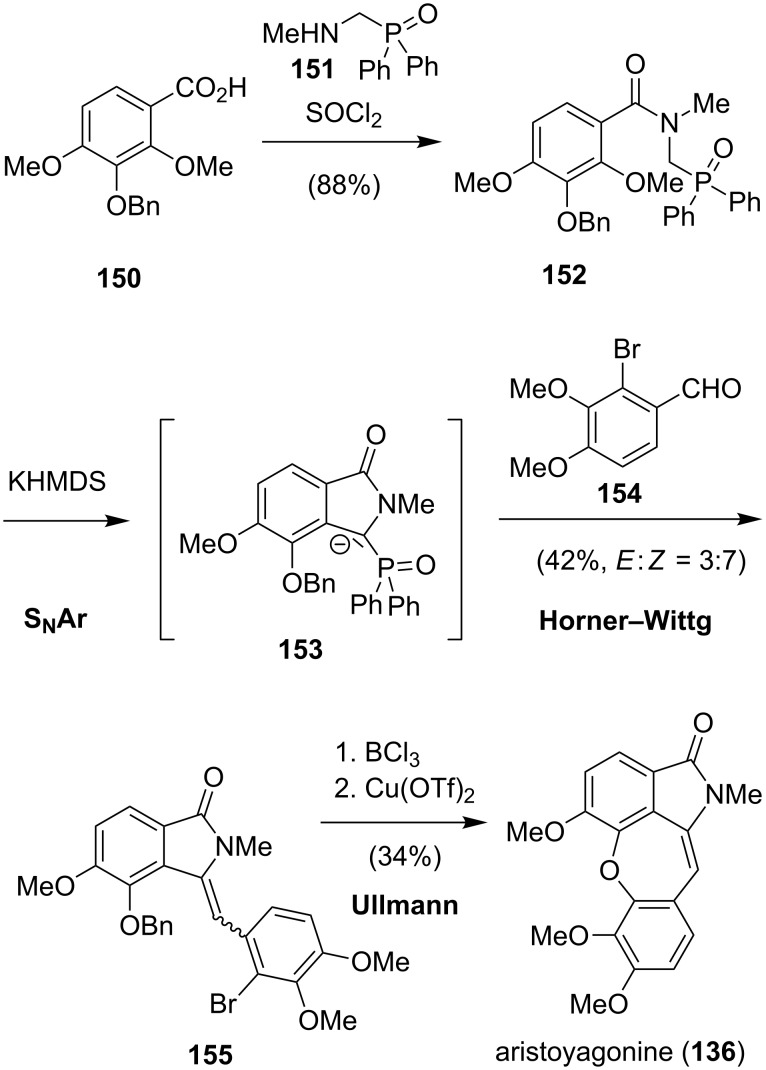
Synthesis of **136** by Couture.

In 2006, another approach to aristoyagonine (**136**) was reported by the group of Couture (Scheme 20) [128]. For the preparation of **136**, their previously developed procedures for the syntheses of aristolactams were adopted [111–112]. The synthesis commenced with the generation of the amide **152** from the coupling of **150** with **151**. Exposure of **152** to potassium bis(trimethylsilyl)amide gave the isoindolinone **153** via an intramolecular addition–elimination reaction. In the presence of the aldehyde **154**, **153** underwent a Horner–Wittig reaction to generate **155** as a 1:2.3 mixture of *E*- and *Z*-isomers. Treatment of **155** with boron trichloride not only liberated the phenol but also isomerized the *E*-double bond to the *Z*-isomer. Finally, a copper(II) triflate-catalyzed Ullmann coupling furnished **136**.

**Figure 8:**
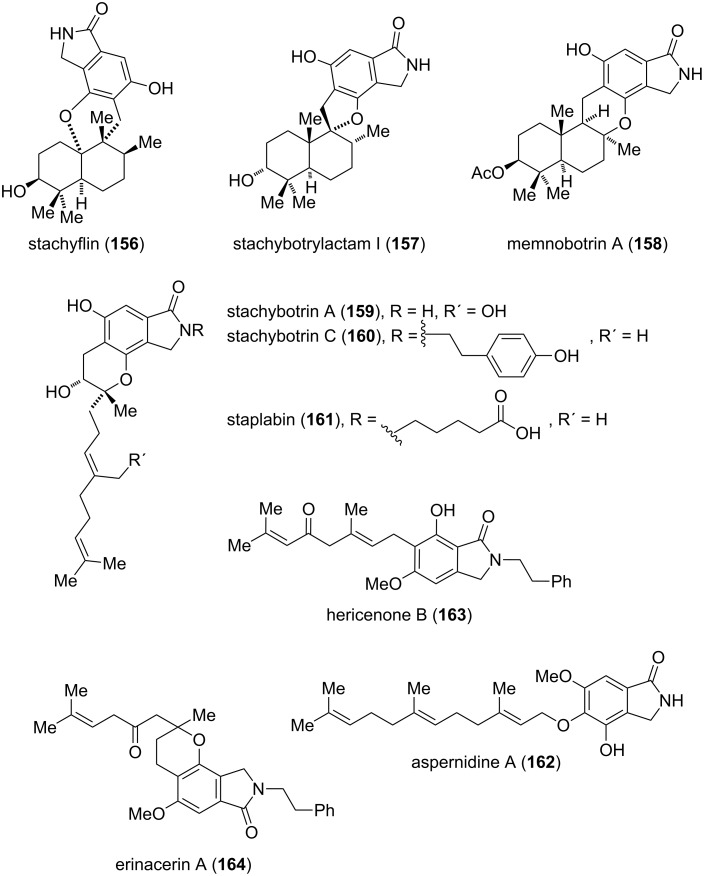
Representative isoindolinone meroterpenoids.

**Meroterpenoids:** The term meroterpenoids describes a family of natural products with a mixed biosynthetic origin, partially derived from terpenoids and polyketides [129]. Several members of this class containing an isoindolinone motif, for instance compounds **156**, **157** and **159**–**161**, can be extracted from *Stachybotrys* fungi (Figure 8) [130–135]. Memnobotrin A (**158**) is found in the closely related *Memnoniella echinata* fungus [136], and hericenone B (**162**) and erinacerin A (**163**) were isolated from the mushroom *Hericium erinaceum* [137–138]. Another member of this family, aspernidine A (**164**), was recently isolated form the fungus *Aspergillus nidulans* by Hertweck [139]. A comprehensive review about meroterpenoids produced by fungi was published by Simpson in 2009 [140]. The isolated molecules display a broad spectrum of interesting biological properties, such as high antiviral activity (**156**) [141], inhibition of the HIV-1 protease (**157**) [132], antibacterial and antifungal activities (**159**–**160**) [133] or neuritogenic properties (**161**) [134]. So far, only a few total syntheses were accomplished and the exact biosynthetic pathways are unknown.

**Scheme 21:**
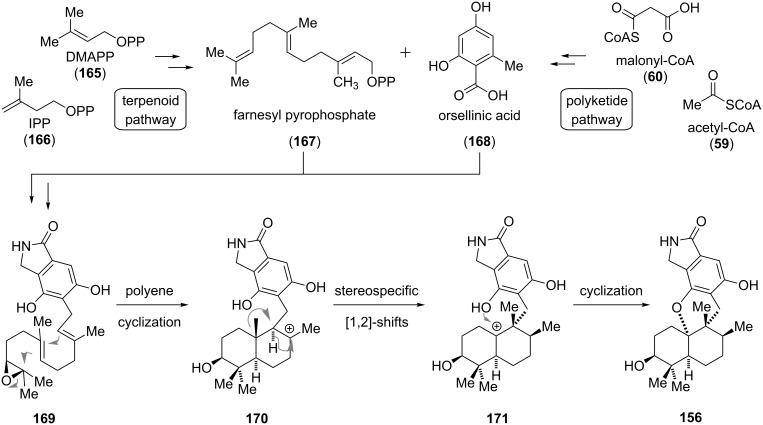
Postulated biosynthetic pathway for the formation of **156** (adopted from George) [143].

The sesquiterpenoid stachyflin (**156**) was first isolated from the fungus *Stachybotrys* sp. RF-7260 by Shionogi & Co., Ltd., Japan, in 1997 [130] and shows outstanding antiviral activity against the influenza A subtype H1N1 (IC_50_ = 3 nM), outperforming current drugs such as amantadine and zanamivir [141]. The putative biosynthesis of **156**, which contains a pentacyclic ring system with a *cis*-fused decalin is depicted in Scheme 21. Farnesyl pyrophosphate (**167**), which originates from the terpenoid pathway through the condensation of dimethylallyl pyrophosphate (DMAPP, **165**) with two units of isopentyl pyrophosphate (IPP, **166**), and orsellinic acid (**168**), which is derived from a fungal iterative type I polyketide pathway [142], are connected to give **169**. This substrate is already poised for a polyene cyclization cascade, which only has to be triggered via activation of the epoxide. After the decalin moiety has formed, two sigmatropic, stereospecific [1,2]-shifts of intermediate **170** provide the tertiary carbocation **171**, which could be trapped by the phenolic alcohol to give stachyflin (**156**) [143].

**Scheme 22:**
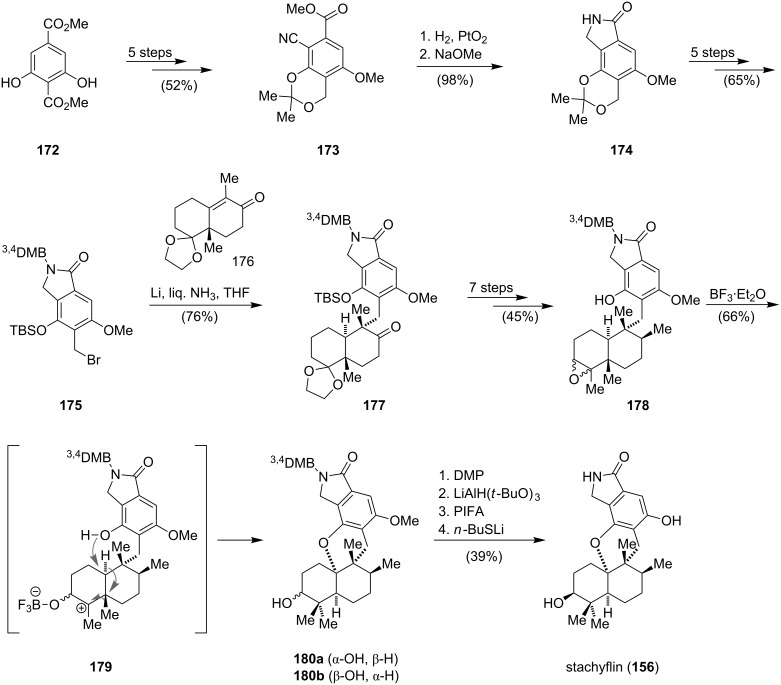
Synthesis of stachyflin (**156**) by Katoh [144].

In 2011, the first enantioselective total synthesis of (+)-stachyflin (**156**) via a Lewis acid induced domino epoxide-opening/rearrangement/cyclization cascade was accomplished by the group of Katoh (Scheme 22) [144]. The synthesis commenced with the conversion of dimethyl 2,6-dihydroxyterephthalate (**172**) to nitrile **173** within five consecutive steps. Hydrogenation and lactam formation of **173** gave isoindolinone **174**, which was converted to bromide **175**. Reductive alkylation of the protected Wieland–Miescher ketone **176** with bromide **175** using Birch conditions gave **177** as a single diastereoisomer. Epoxide **178** could be prepared in seven steps from **177**, but was obtained as a mixture of inseparable diastereoisomers. The Lewis acid induced key step, a domino epoxide-opening/rearrangement/cyclization cascade, most likely proceeded in a stepwise manner via intermediate **179**. Activation of the epoxide with boron trifluoride etherate induces the planned sigmatropic [1,2]-methyl and [1,2]-hydride shifts to generate the tertiary carbocation at the ring junction. This is then trapped by the phenol to give the pentacyclic compounds **180a** and **180b**. The former diastereoisomer could be converted to **180b** via inversion of the secondary alcohol. Cleavage of the methyl ether and the 3,4-dimethoxybenzyl (^3,4^DMB) group of **180b** led to (+)-stachyflin (**156**). The synthesis proceeded in 25 steps with an overall yield of 2.6%.

**Figure 9:**
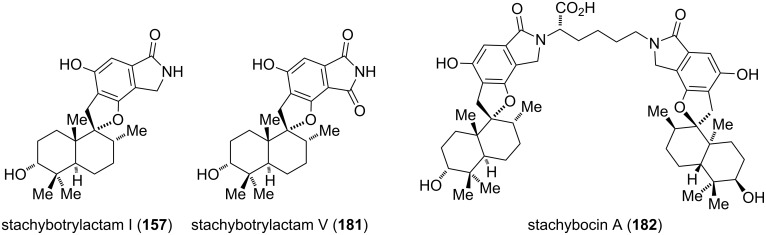
Selected examples of spirodihydrobenzofuranlactams.

Another novel class of isoindolinone-containing natural products, categorized as spirodihydrobenzofuranlactams or stachybotrylactams (**157**, **181**, **182**), was isolated from different *Stachybotrys* species by Jarvis and Roggo between 1995 and 1996 (Figure 9) [131–132]. These compounds are antagonists of endothelin and display strong immunosuppressant activities. The pseudosymmetric dimer stachybocin A (**182**), which was isolated by Ogawa in 1995 [145], constitutes the most potent representative [132]. Oxidation of the isoindolinone unit of **157** gives the relatively scarce phthalimide motif, which is present in stachybotrylactam V (**181**).

**Scheme 23:**
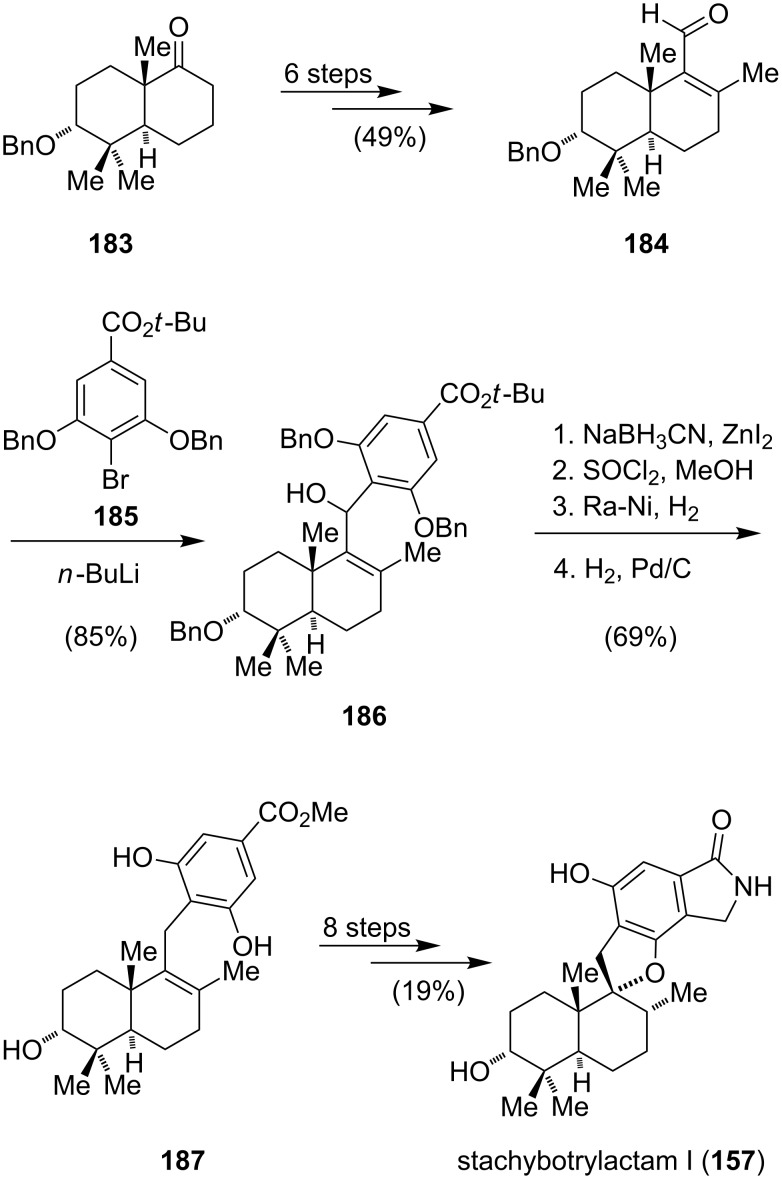
Synthesis of stachybotrylactam I (**157**).

To date, only one enantioselective total synthesis of spirodihydrobenzofuranlactam I **157** has been published (Scheme 23) [146]. The synthesis carried out by the group of Guo commenced with *trans*-decalone **183**, which was derived from the Wieland–Miescher ketone. For the conversion of **183** to the α,β-unsaturated aldehyde **184** six steps were necessary. Halogen–metal exchange of **185** with *n*-butyllithium followed by the addition of **184** gave the carbinol **186** in good yield. Removal of the benzyl groups, cleavage of the *tert*-butyl group with concomitant formation of the methoxy ester (COO*t*-Bu → COOMe), and global deprotection gave **187**. Acid-catalyzed (Amberlyst 15) spiroannulation afforded a 1.7:1 mixture of the benzofuran and the benzopyran (90% overall conversion). For the installation of the isoindolinone, a seven-step sequence similar to the one described for stachyflin (**156**) was used (Scheme 23). The arene appendage was desymmetrized via monobromination and cross coupling with copper cyanide provided a nitrile. Hydrogenation and lactam formation under basic conditions completed the synthesis of stachybotrylactam I (**157**).

**Scheme 24:**
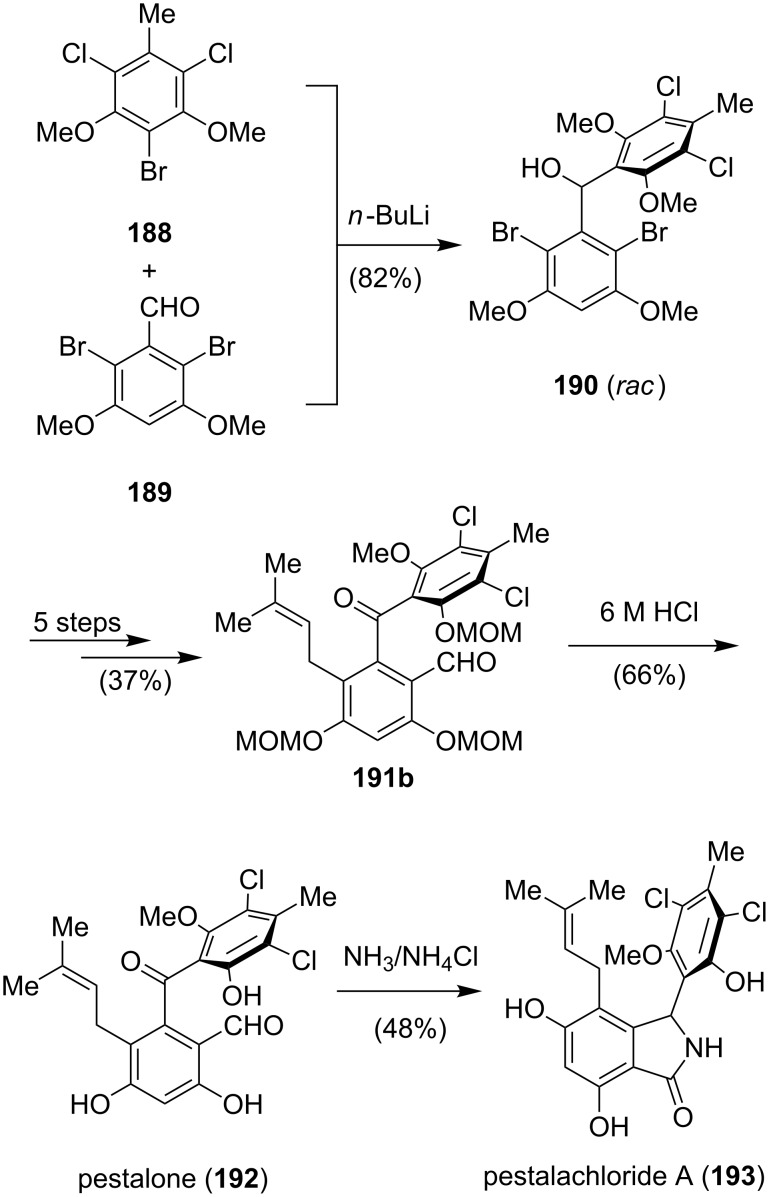
Synthesis of pestalachloride A (**193**) by Schmalz.

**Chlorinated isoindolinone alkaloids:** Pestalachloride A (**193**) was isolated from the plant endophytic fungus *Pestalotiopsis adusta* as a racemate and displays potent antifungal activities against *Fusarium culmorum* (IC_50_ = 0.89 µM) [147]. Pestalone (**192**), a natural product and synthetic precursor of **193** shows strong antibiotic activity against methicillin-resistant *Staphylococcus aureus* (MRSA, MIC = 84 nM) and vancomycin-resistant *Enterococcus* (VRE, MIC = 178 nM) [148]. The structural similarity between pestalachloride A (**193**) and pestalone (**192**), which both contain the same prenylated polyketide core imply a direct biosynthetic relationship [147]. The reaction of **192** with an equivalent of ammonia gives a cyclic iminohemiaminal, which first tautomerizes to the hydroxy isoindole and then to the isoindolinone **193**. This biosynthetic transformation was also used in the total synthesis of **193** by Schmalz (Scheme 24) [149]. Reaction of lithiated **188** (three steps from commercially available 5-methylresorcinol) with the aldehyde **189** (double bromination of 3,5-dimethoxybenzaldehyde) gave the racemic alcohol **190**.

**Scheme 25:**
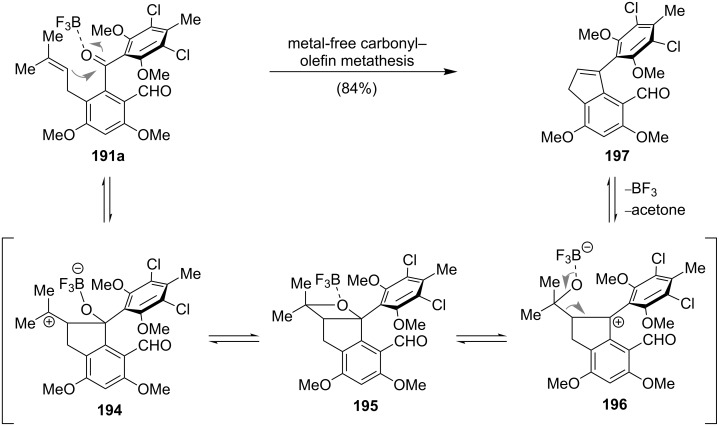
Proposed mechanism for the BF_3_-catalyzed metal-free carbonyl–olefin metathesis [149].

Oxidation to the benzophenone, installation of the prenyl side chain and introduction of the formyl group was accomplished within five steps to give **191a** (Scheme 25). The envisioned deprotection of **191a** using Lewis acidic conditions led to an unexpected and unprecedented metal-free carbonyl–olefin metathesis. Coordination of boron trifluoride etherate to **191a** promotes the formation of the tertiary carbenium ion **194**, which isomerizes via the oxetane intermediate **195** to the benzylic cation **196**. Expulsion of acetone then yields indene **197** in excellent yield. Avoiding this side reaction could only be achieved by exchanging the methyl ether protecting group for a more labile methoxymethyl ether at the benzophenone stage. The following prenylation and formylation proceeded smoothly under the same conditions to give **191b** (Scheme 24). Cleavage of the MOM ethers afforded pestalone (**192**), which could be converted into **193** in a single step by treatment of **192** with ammonia in aqueous ammonium chloride solution (pH 8).

**Scheme 26:**
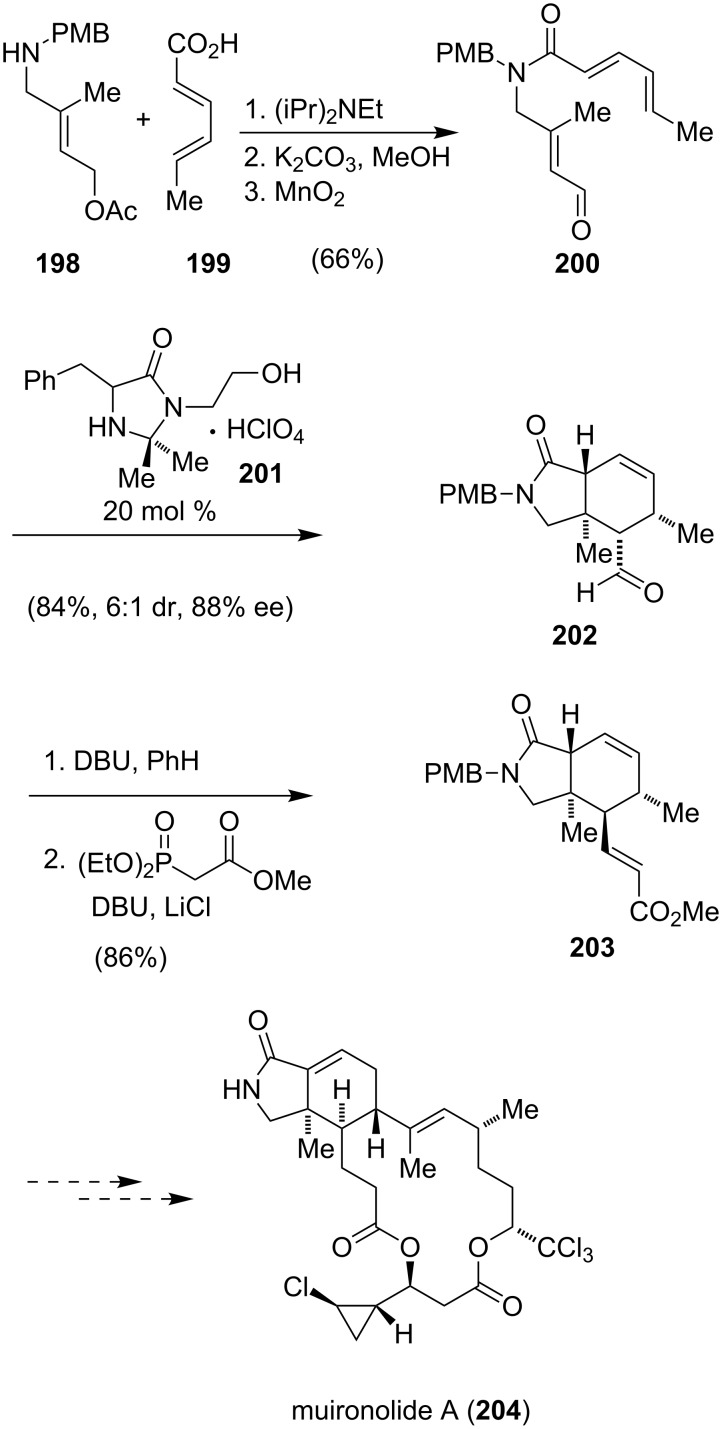
Preparation of the isoindoline core of muironolide A (**204**).

Muironolide A (**204**) was isolated by Molinski from the marine sponge *Phorbas* sp. in 2009 [150]. The molecular framework of this unique natural product has a hexahydro-1*H*-isoindolinone-triketide, a *trans*-2-chlorocyclopropane and a trichlorocarbinol ester. A first biological evaluation showed that **204** displays antifungal activity. However, only 90 µg could be isolated and further biological screening was not possible [151]. Several sponge-derived macrolides have proven to be effective cytotoxic agents [152–153] and one could speculate that muironolide A (**204**) shares this potential. A first attempt to access **204** in the laboratory was reported by Molinski shortly after the isolation (Scheme 26) [151]. Coupling of the dienophile precursor **198** with sorbic acid (**199**) gave a tertiary amide, which was converted to **200** via a reduction–oxidation sequence. The asymmetric intramolecular Diels–Alder reaction was then catalyzed by Kristensens’ catalyst (**201**) to give **202**. A base-promoted epimerization of the aldehyde and a Horner–Wadsworth–Emmons reaction furnished **203**, the most advanced intermediate reported so far.

Just recently, the group of Zakarian reported another approach to the fully elaborated isoindolinone core of **204** [154].

**Scheme 27:**
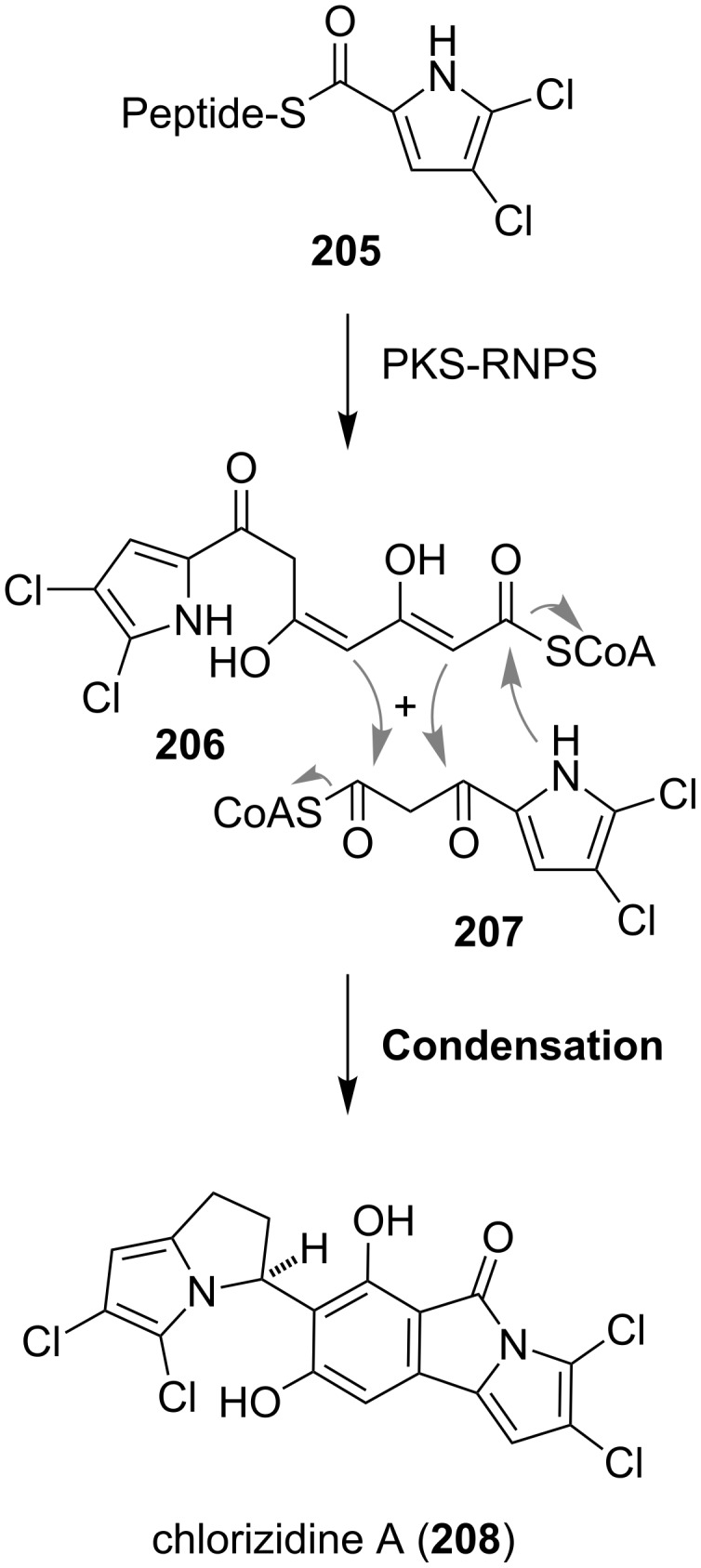
Proposed biosynthesis of **208**.

Chlorizidine A (**208**) (Scheme 27), a cytotoxic metabolite from a marine *Streptomyces* species shows an unprecedented 5*H*-pyrrolo[2,1-*a*]isoindolinone ring system, which is connected to a dichlorinated pyrrolizine [155]. Chlorizidine A (**208**) and its semisynthetic derivatives display significant cytotoxic activities against various human cancer cell lines. The IC_50_ value of **208** against the colon cancer cell line HCT-116 was determined to be in the micromolar range (3.2–4.9 μM) [155]. A first biosynthesis was postulated on the basis of the structural similarity between **208** and marinopyrrole A, another secondary metabolite derived from a marine *Streptomyces* species [156]. The common biosynthetic precursor **206** stems from a mixed nonribosomal peptide synthetase (NRPS)/polyketide synthase (PKS) pathway. The amino acid proline is first oxidized and chlorinated by an FADH_2_-dependent halogenase to give **205**, which after extension by the polyketide synthase gives the polyketides **206** and **207**, respectively. The intermolecular condensation of these two components yields chlorizidine A (**208**) [155].

**Scheme 28:**
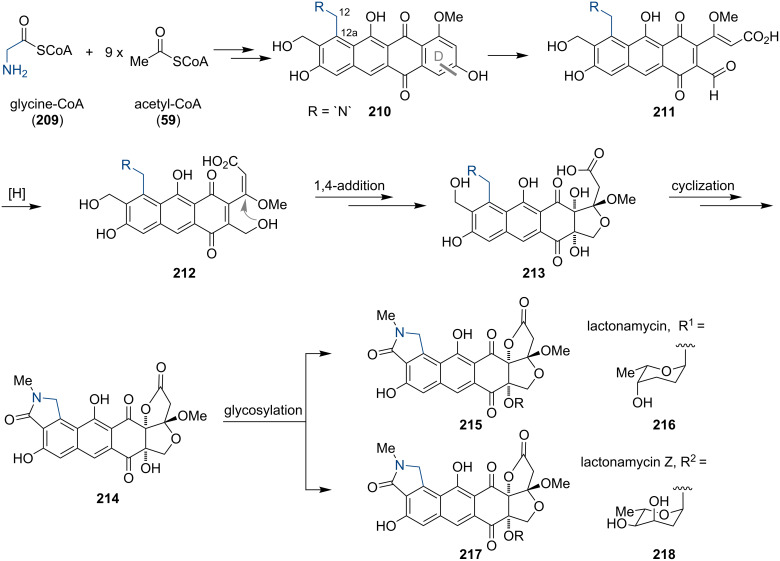
Model for the biosynthesis of **215** and **217**.

**Anthraquinone-type alkaloids:** Lactonamycin (**215**) and lactonamycin Z (**217**) were isolated from *Streptomyc*es *rishiriensis* and *Streptomyces anglieri* in 1996 and 2003 [157–158]. Both compounds are potent antibacterial agents against Gram-positive bacteria, including methicillin-resistant *Staphylococcus aureus* (MRSA) and vancomycin-resistant *Enterococcus* (VRE) [159]. Beyond that, the modest cytotoxic activity of **215** and **217** against human cancer cell lines makes them interesting lead components for drug discovery. The IC_50_ values of **215** for different leukemia cell lines range from 0.11–0.22 µM. For **217**, an IC_50_ value of 0.32 µM against gastric adenocarcinoma was observed [157–158]. The hexacyclic aglycone of **215** and **217**, lactonamycinone (**214**), consists of a densely oxygenated fused hydrofuran–hydrofuranone and a naphta[*e*]isoindole ring system. The core of lactonamycin (**215**) is decorated with the 2-deoxysugar α-L-rhodinopyranose (**216**), whereas α-L-2,6-dideoxyribopyranose (**218**) is found in **217**. The structure of the aglycone **214** is related to tetracenomycins, a family of tetracyclic aromatic polyketides, which are produced by several *Streptomyces* species [159]. Biosynthetic investigations revealed a striking similarity of the gene cluster responsible for the biogenesis of lactonamycinone (**214**) to those clusters found in tetracenomycin-producing bacteria. Cloning experiments and incorporation of various labeled precursors resulted in a first hypothesis for the biogenesis of **215** and **217** (Scheme 28) [159]. This led to the assumption that the aglycone **214** is assembled from nine acetate units and glycine, respectively *N*-methylglycine derivative, which contributes both carbon atoms and the nitrogen to the skeleton of **210** (position 12 and 12a). The latter are uncommon starter units in a type II polyketide assembly line [160]. Upon oxidative cleavage of the D-ring of the putative tetracenomycin-like intermediate **210**, the aldehyde **211** is reduced to the corresponding alcohol **212**. Acetalization and introduction of the 1,2-*cis*-diol moiety furnishes **213**. Sequential formation of the lactone and the isoindolinone generates **214**. A final glycosylation step leads to lactonamycin (**215**) and lactonamycin Z (**217**).

**Scheme 29:**
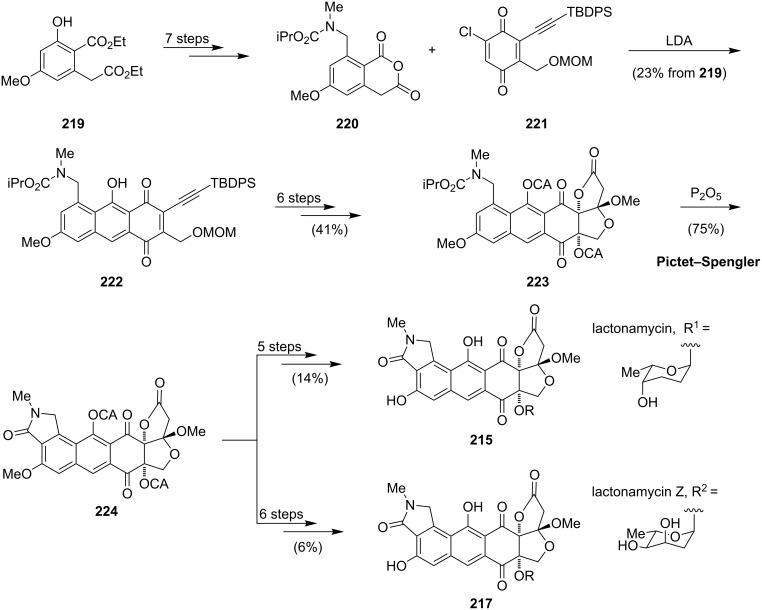
Synthesis of lactonamycin (**215**) and lactonamycin Z (**217**).

The remarkable structure and the promising biological activities have attracted many synthetic groups. Several total syntheses of **215** and **217** were reported in recent years [161] (and references therein). The group of Danishefsky was the first to report a diastereoselective synthesis of lactonamycinone (**214**) employing a Diels–Alder reaction [162–163]. In 2010, Tastuta completed the first total synthesis of lactonamycin (**215**) by using a sequential conjugate addition, a stereoselective glycosylation reaction and a Michael–Dieckmann-type cyclization [164]. The recently published total synthesis of lactonamycin (**215**) and lactonamycin Z (**217**) by Saikawa and Nakata is based on a late-stage glycosylation strategy (Scheme 29). This enables the specific variation of the sugar components and gives access to various lactonamycin derivatives [161].

Starting from alcohol **219**, the diene precursor **220** could be prepared in seven steps. The following Diels–Alder reaction with chloroethynylquinone **221**, which was synthesized according to a known procedure [165] proceeded in a highly regioselective manner to give antharaquinone **222**. Introduction of the fused hydrofuran–hydrofuranone moiety was accomplished via deprotection, palladium-catalyzed cyclization–methoxycarbonylation [166] and an acid-catalyzed lactone formation to afford **223**. For the generation of the isoindolinone via a Bischler–Napieralski reaction, a chloroacetyl (CA)-protected phenol was essential to avoid competing carbamate formation of the starting material. Upon exposure of **223** to phosphorus pentoxide, the desired isoindolinone **224** was formed in 75% yield. Deprotection led to lactonamycinone (**214**), which was the substrate for a ytterbium triflate-catalyzed glycosylation to give **215** and **217**, respectively.

**Figure 10:**
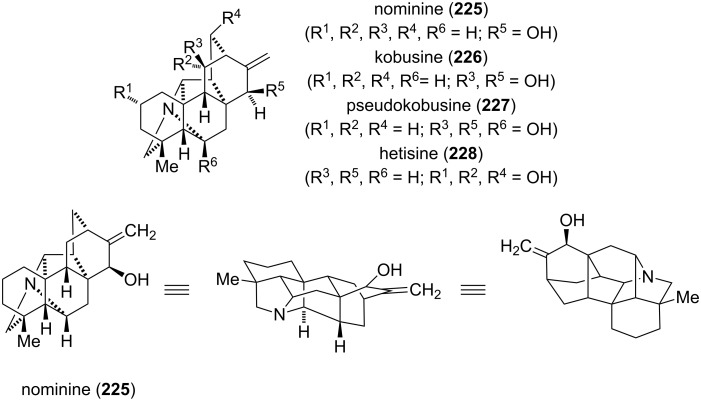
Hetisine alkaloids **225**–**228**.

**Miscellaneous:** At first glance, the hetisine alkaloids do not show any structural similarities with the compound classes described before. However, though lacking an “intact” isoindole core, one can spot the corresponding perhydro motif. Since their first discovery more than 70 years ago, more than 100 different hetisine alkaloids have been isolated from different species of the plants *Aconitum* and *Delphinium*, and to a lesser extend from *Rumex*, *Consolida* and *Spiraea* [167–168]. These plants have been widely used in traditional herbal medicine and further pharmacological studies revealed that hetisine alkaloids are highly bioactive compounds [167–168]. The diverse spectrum includes vasodilatoring, anti-arrhythmic, immunomodulating and analgesic activities. Hetisines can be classified, according to Wang and Liang, as C_20_-diterpenoid alkaloids, which belong to the family of atisane alkaloids [167]. The hetisines comprise a heptacyclic core and are structurally the most complex members derived from the atisine skeleton. The most prominent representatives, nominine (**225**) [169–170], kobusine (**226**) [171–172], pseudokobusine (**227**) [173–174] and hetisine (**228**) [174–176] are depicted in Figure 10.

**Scheme 30:**
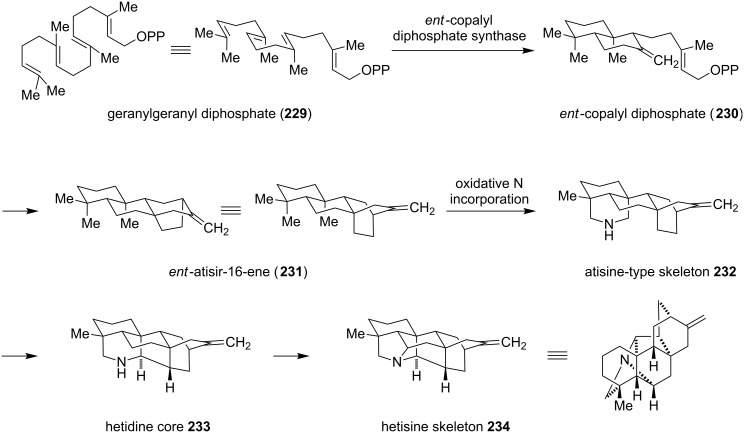
Biosynthetic proposal for the formation of the hetisine core [167].

The putative biosynthetic pathway for the formation of the hetisine alkaloids is derived from phytochemical data and basic biochemical transformations (Scheme 30) [167]. Cyclization of geranylgeranyl pyrophosphate (**229**), involving the *ent*-copalyl diphosphate synthase, could give *ent*-copalyl diphosphate (**230**). After loss of the pyrophosphate group and double cyclization, a 1,3-hydride shift and Wagner–Meerwein rearrangement leads to the naturally occurring *ent*-atisir-16-ene (**231**). Oxidative incorporation of nitrogen, which might be derived from β-aminoethanol, generates the atisine-type skeleton **232**. Oxidative C–C-bond formation gives the hetidine core (**233**) and C–N-bond formation the hetisine skeleton (**234**).

**Scheme 31:**
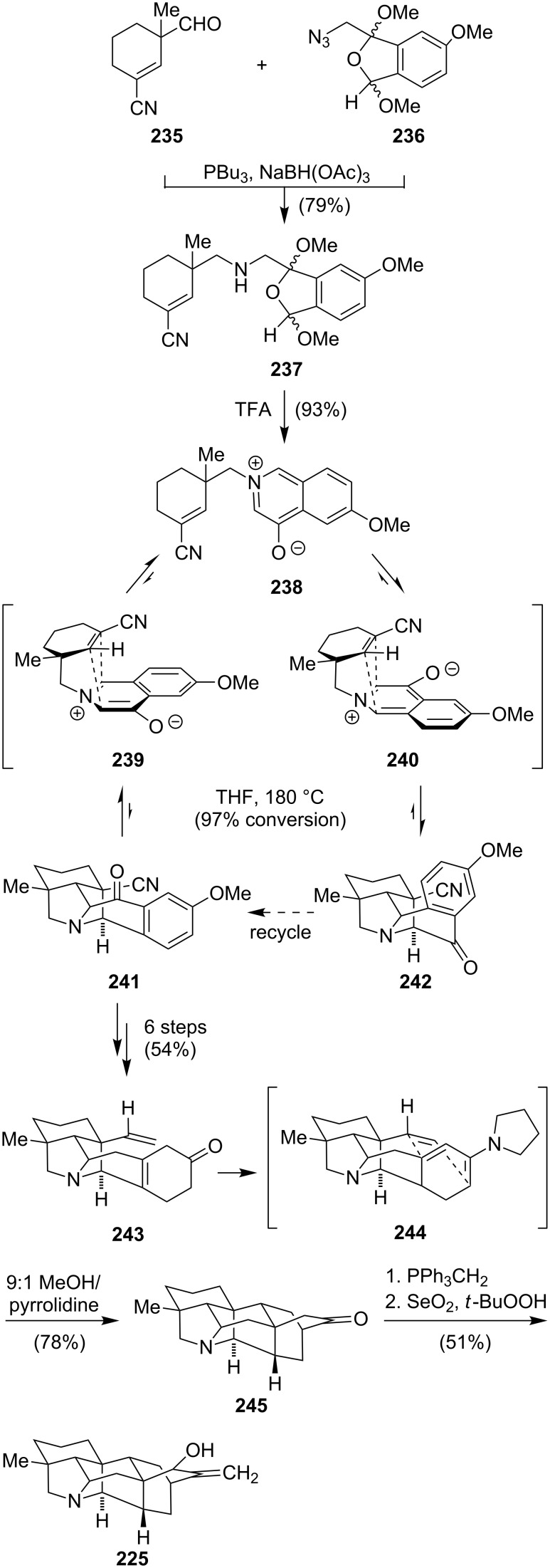
Synthesis of nominine (**225**).

The first total synthesis of a hetisine-type alkaloid was accomplished by Muratake and Natsume in 2004 [177]. In their seminal work, (±)-nominine (**225**) was synthesized within 40 steps and 0.15% overall yield. Two years later, a more concise and efficient access to (±)-nominine (**225**), featuring a oxidoisoquinolinium-1,3-dipolar cycloaddition and a dienamine-Diels–Alder reaction, was accomplished by Gin (Scheme 31) [178]. Coupling of **235** and **236**, both synthesized within three steps from simple starting materials, via a Staudinger–aza-Wittig reaction gave amine **237** as a mixture of four diastereoisomers. Conversion into 4-oxido-isoquinolinium betaine **238** could be achieved by an acid-catalyzed methanol extrusion and isomerization. Betaine **238** served as the substrate for the following 1,3-aza-dipolar cycloaddition. Carrying out the reaction at 180 °C in tetrahydrofuran provided a separable mixture of pyrrolidine isomers **241** and **242** (1:3.6). The undesired cycloadduct **242** could be equilibrated to **241** due to the reversibility of the reaction. Conversion to the β,γ-cyclohexenone **243** was accomplished within 6 consecutive steps. Generation of the dienamine **244** with pyrrolidine in methanol at 60 °C triggered an intramolecular Diels–Alder reaction to provide the full carbon skeleton **245**. The final transformations of the synthesis involved a Wittig olefination of the ketone and a diastereoselective allylic oxidation to provide (±)-nominine (**225**). In 2008, the same group reported the first total synthesis of (+)-nominine by introducing the desired stereochemical information to the nitrile **235** [179].

## Conclusion

The outlined syntheses of natural products containing the uncommon isoindole skeleton are still far away from being ideal. Low yielding multistep strategies dominate for more complex molecules and often cannot provide the amounts necessary for further investigations. Several biologically active members are still precluded from extensive biological studies due to their low abundance in nature. This and the fact, that many compounds are underexplored offers plenty opportunities for the development of innovative chemical transformations. The biosynthesis of many isoindole natural products is still uncertain and has yet to be unraveled. The presented examples could serve as an inspiration for the development of novel synthetic methods, new biosynthetic insights and drug development. Considerably more discoveries remain to be uncovered through exciting projects.
